# Defective Mitochondrial Quality Control during Dengue Infection Contributes to Disease Pathogenesis

**DOI:** 10.1128/jvi.00828-22

**Published:** 2022-10-05

**Authors:** Bharati Singh, Kiran Avula, Shamim Akhtar Sufi, Nahid Parwin, Sayani Das, Mohd Faraz Alam, Subhashish Samantaray, Leelakrishna Bankapalli, Alankrita Rani, Kokavalla Poornima, Biswajita Prusty, Tareni P. Mallick, Shubham K. Shaw, Hiren Dodia, Shobhitendu Kabi, Trupti T. Pagad, Sriprasad Mohanty, Gulam Hussain Syed

**Affiliations:** a Institute of Life Sciences, Bhubaneswar, Odisha, India; b Kalinga Institute of Information and Technology, Bhubaneswar, Odisha, India; c Regional Centre for Biotechnology, Faridabad, Haryana, India; d Department of Medicine, Institute of Medical Sciences & SUM Hospital, Bhubaneswar, Odisha, India; e SCB Medical College, Cuttack, Odisha, India; University of Southern California

**Keywords:** autophagy, dengue virus, inflammation, mitochondria, mitochondrial homeostasis, mitochondrial quality control, mitophagy, necrosis, mt-DNA

## Abstract

Mitochondrial fitness is governed by mitochondrial quality control pathways comprising mitochondrial dynamics and mitochondrial-selective autophagy (mitophagy). Disruption of these processes has been implicated in many human diseases, including viral infections. Here, we report a comprehensive analysis of the effect of dengue infection on host mitochondrial homeostasis and its significance in dengue disease pathogenesis. Despite severe mitochondrial stress and injury, we observed that the pathways of mitochondrial quality control and mitochondrial biogenesis are paradoxically downregulated in dengue-infected human liver cells. This leads to the disruption of mitochondrial homeostasis and the onset of cellular injury and necrotic death in the infected cells. Interestingly, dengue promotes global autophagy but selectively disrupts mitochondrial-selective autophagy (mitophagy). Dengue downregulates the expression of PINK1 and Parkin, the two major proteins involved in tagging the damaged mitochondria for elimination through mitophagy. Mitophagy flux assays also suggest that Parkin-independent pathways of mitophagy are also inactive during dengue infection. Dengue infection also disrupts mitochondrial biogenesis by downregulating the master regulators PPARγ and PGC1α. Dengue-infected cells release mitochondrial damage-associated molecular patterns (mtDAMPs) such as mitochondrial DNA into the cytosol and extracellular milieu. Furthermore, the challenge of naive immune cells with culture supernatants from dengue-infected liver cells was sufficient to trigger proinflammatory signaling. In correlation with our *in vitro* observations, dengue patients have high levels of cell-free mitochondrial DNA in their blood in proportion to the degree of thrombocytopenia. Overall, our study shows how defective mitochondrial homeostasis in dengue-infected liver cells can drive dengue disease pathogenesis.

**IMPORTANCE** Many viruses target host cell mitochondria to create a microenvironment conducive to viral dissemination. Dengue virus also exploits host cell mitochondria to facilitate its viral life cycle. Dengue infection of liver cells leads to severe mitochondrial injury and inhibition of proteins that regulate mitochondrial quality control and biogenesis, thereby disrupting mitochondrial homeostasis. A defect in mitochondrial quality control leads to the accumulation of damaged mitochondria and promotes cellular injury. This leads to the release of mitochondrial damage-associated molecular patterns (mt-DAMPs) into the cell cytoplasm and extracellular milieu. These mt-DAMPs activate the naive immune cells and trigger proinflammatory signaling, leading to the release of cytokines and chemokines, which may trigger systemic inflammation and contribute to dengue disease pathogenesis. In correlation with this, we observed high levels of cell-free mitochondrial DNA in dengue patient blood. This study provides insight into how the disruption of mitochondrial quality control in dengue-infected cells can trigger inflammation and drive dengue disease pathogenesis.

## INTRODUCTION

Dengue is among the most dangerous and rapidly emerging arthropod-borne human pathogens. It is widespread throughout tropical and subtropical countries. With nearly 4 billion people living in regions at risk of dengue infection, dengue has rapidly emerged as a global health burden. Dengue is transmitted by female Aedes aegypti and Aedes albopictus mosquitoes, which also serve as vectors for other major human pathogens such as chikungunya, yellow fever, and zika viruses (1). Dengue virus (DENV) belongs to the *Flavivirus* genus within the Flaviviridae family, and four distinct but closely related serotypes DENV-1, DENV-2, DENV-3, and DENV-4 circulate worldwide. The 11-kb single-stranded +ve RNA genome of dengue virus encodes a single polyprotein, which is cleaved by host and viral proteases into three structural (capsid, precursor membrane [prM], and envelope) and seven nonstructural proteins (NS1, NS2A, NS2B, NS3, NS4A, NS4B, and NS5) (2). Nearly 50 to 100 million infections are reported annually, with most patients manifesting subclinical or flu-like febrile illness (2). Some patients develop dengue with severe complications such as mucosal bleeding, abdominal discomfort, hepatomegaly, thrombocytopenia, and pleural effusion, leading to dengue hemorrhagic fever (DHF), which may manifest into dengue shock syndrome (DSS), which is associated with severe bleeding, plasma leakage, and/or organ impairment (3). There are no specific therapeutic strategies against dengue, and treatment is limited to symptomatic and supportive care. The Dengvaxia vaccine, licensed for use in some dengue-endemic countries, does not have optimal efficacy against all serotypes and is considered unsafe in seronegative individuals (4). This warrants the need to precisely understand the molecular events that are associated with the disease.

Induction of the immune responses and high levels of proinflammatory cytokines are implicated in severe dengue. However, our understanding of the molecular cues that trigger the increased activation of immune cells, resulting in exacerbated immune responses or cytokine storms during dengue infection, is still rudimentary. Factors exacerbating the inflammatory responses have been associated with an increased risk of severe disease (4). In general, the effect of the viruses on various cellular components in host cells contributes to the onset of disease pathogenicity. Mitochondria is a dynamic organelle that plays a significant role in cellular metabolism, antiviral signaling, and inflammatory signaling (5). Many viruses target mitochondria, as their disruption can facilitate a cellular environment conducive to viral dissemination (5). Previous studies indicate that DENV promotes mitochondrial dysfunction (6) and perturbs mitochondrial dynamics, with some groups suggesting enhanced mitochondrial fusion (7, 8) and other groups suggesting enhanced fission (9). Damaged mitochondria have a deleterious effect on cellular health and hence have to be rapidly cleared to maintain cellular homeostasis (10, 11). Cells have evolved multiple mechanisms to ensure mitochondrial quality control and maintain a requisite number of functional mitochondria. These mechanisms coordinate to eliminate damaged mitochondria or mitochondrial proteins and renew components through biogenesis, resulting in a rapid mitochondrial turnover. The clearance of the damaged mitochondria is accomplished by mitochondrial-selective autophagy (mitophagy) (10, 11). The PINK1-Parkin pathway is the most characterized mechanism of mitophagy, whereby the damaged mitochondria are flagged for elimination by PINK1 (PTEN-induced putative kinase 1), a serine-threonine kinase, and Parkin, a cytosolic E3 ubiquitin ligase (12, 13). However, many recent pieces of evidence suggest that Parkin-independent mechanisms of mitophagy are also operative under different physiological and pathological contexts (10, 11). Impaired clearance of damaged mitochondria results in their accumulation, which leads to the generation of high levels of reactive oxygen species (ROS) that can injure healthy mitochondria and start a vicious loop of ROS generation and mitochondrial damage, eventually resulting in oxidative damage of major cellular components and cell death (5). Accumulation of damaged mitochondria causes mitochondrial damage-associated molecular patterns (mt-DAMPs) to be released into the cytosol or extracellular environment, which can trigger the production of proinflammatory mediators and exacerbate the host inflammatory response (14, 15).

Many DENV proteins have been shown to localize to the mitochondria and alter host mitochondrial dynamics and functions and interfere with mitochondrial antiviral signaling to promote viral processes (7–9). However, there is a lack of understanding of how dengue affects the host’s mitochondrial homeostasis and quality control and the significance of this in dengue disease pathogenesis. In this study, we show how DENV disrupts mitochondrial quality control through the inhibition of mitochondrial-selective autophagy and mitochondrial biogenesis and delineate the mechanisms involved. We show that the dengue-infected hepatic cells release mitochondrial DNA (mt-DNA) into the extracellular milieu and induce proinflammatory signaling. We observed a strong correlation between the levels of cell-free mt-DNA in dengue patient samples and the degree of thrombocytopenia. Overall, our study demonstrates how defective mitochondrial quality control in DENV-infected cells can lead to the release of mt-DAMPs into circulation and trigger the activation of proinflammatory signaling and cytokine production.

## RESULTS

### Dengue infection leads to profound levels of mitochondrial injury in the hepatic cells.

**FIG 1:**
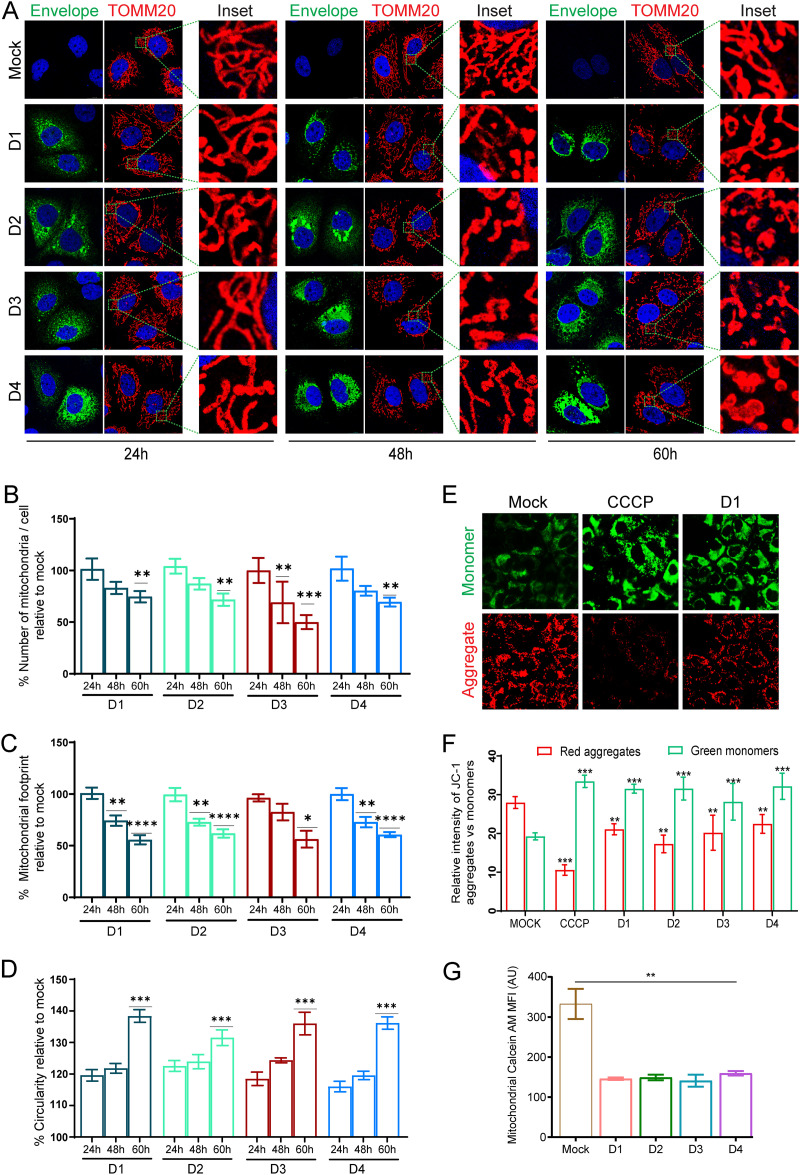
Dengue infection induces mitochondrial injury in Huh7 cells. (A) Confocal microscopy images of Huh7 cells infected with various dengue virus serotypes at the indicated time points. The cells were immunostained with serotype-specific anti-envelope and mitochondrion-specific anti-TOMM20 antibodies. Nuclei were counterstained with DAPI. Scale bar = 10 μm. Around 30 to 40 cells per condition were quantified for the various features of the mitochondria, such as number, length, and circularity. (B to D) The quantification is depicted as the percentage of mock-infected cells for average mitochondrial number per cell (B), mitochondrial footprint (C), and circularity (D). (E) Fluorescence microscopy images of the dengue serotype 1 (D1)-infected, mock-infected, and CCCP-treated Huh7 cells stained with the mitochondrial membrane potential specific dye JC1. (F) Relative fluorescence intensity of JC-1 aggregates (red) versus monomers (green) in mock-infected, CCCP-treated, and specific dengue serotype-infected Huh7 cells. (G) Graph depicting the status of mitochondrial permeability transition pore (mPTP) opening represented by the mean fluorescence intensity (MFI) of the mitochondrial calcein retained in mitochondria in mock-infected versus dengue serotype-infected cells at 48h postinfection. Data presented are the mean ± standard error of the mean (SEM) of three independent experiments. Statistical analysis was done using one-way ANOVA (B, D, and G) or two-way ANOVA (F). *, *P < *0.05; **, *P < *0.01; ***, *P < *0.001; ****, *P < *0.0001.

To determine the impact of dengue infection on mitochondrial homeostasis, we first determined the effect of dengue on host mitochondrial morphology during the entire course of infection. Confocal imaging of the human liver Huh7 cells, infected with 1 multiplicity of infection (MOI) of the four serotypes of DENV suggested that the DENV-infected cells display a normal mitochondrial network very similar to that of the mock-infected cells at 24 h postinfection (hpi). However, at 48 hpi a significant number of mitochondria showed round and swollen morphology, and at 60 hpi most of the mitochondria displayed highly swollen morphology and detachment from the reticular mitochondrial network (Fig. 1A). Quantitation of the mitochondrial number and morphological features using the ImageJ macro Mitochondria Analyzer clearly indicated a time-dependent decline in the overall number of mitochondria in DENV-infected cells (Fig. 1B) associated with a decline in the overall mitochondrial footprint (Fig. 1C) and an increase in the average circularity or mitochondrial swelling (Fig. 1D) during the course of infection. In agreement with previous studies (7, 8), we did observe a few highly elongated tubular mitochondrial filaments in DENV-infected cells that gradually declined in number at the later time point (see Fig. S1A and B in the supplemental material). The high degree of mitochondrial swollenness indicated severe mitochondrial injury in DENV-infected Huh7 cells. Fluorescence microscopy using mitochondrial membrane potential-specific JC-1 fluorescent dye clearly showed significantly high levels of green JC-1 monomers in DENV-infected cells, almost to the extent observed in mitochondrial decoupler (CCCP)-treated mock cells, indicating a loss of mitochondrial membrane potential in dengue-infected cells. In contrast, the mock-infected cells displayed high levels of the red JC-1 aggregates (Fig. 1E and F). A mitochondrial permeability transition pore (mPTP) assay showed a high degree of quenching of mitochondrial calcein fluorescence in DENV-infected cells, indicating the opening of mPTP in DENV-infected cells (Fig. 1G). mPTP opening leads to generation of mitochondrial ROS, which was confirmed by the presence of high levels of mitochondrial superoxide in DENV-infected cells at 48 hpi (Fig. S1C). Apart from Huh7 cells, we have also looked into the effect of DENV on the mitochondria in other epithelial cell lines, such as A549 cells (lung epithelial cell line) and HEK cells (human embryo kidney cell line). Similar to observations in Huh7 cells, we observed a decline in the total number of mitochondria and mitochondrial footprints associated with an increase in mitochondrial circularity during the course of infection, in both A549 and HEK cells infected with DENV serotype 2 in comparison to mock-infected cells (Fig. S2A and B). Both cell lines also generated high levels of mitochondrial ROS, which was confirmed by the presence of high levels of mitochondrial superoxide at 48 hpi (Fig. S2C and D). We then investigated the status of proteins that govern mitochondrial dynamics. In agreement with previous studies (7–9), we observed that dengue perturbs both the mitochondrial fission and fusion machinery during the course of infection. DENV downregulated the expression levels of the mitochondrial fission protein DRP1 and its S616 phosphorylated active form within 48 hpi, whereas the decline in the mitochondrial outer membrane fusion proteins, mitofusin 1 and 2 was more evident at a later time postinfection (Fig. S3A and B). We did not observe a significant change in the expression status of the mitochondrial inner membrane fusion protein OPA1 (Fig. S3B). In line with the decline in expression of DRP1, we also observed a decline in the expression of mitochondrial fission factor (MFF), an integral membrane protein of the mitochondrial outer membrane that plays a major role in the recruitment of DRP1 to mitochondria (Fig. S3A). We did not observe any significant change in the expression status of Miro 1 (Fig. S3A), a mitochondrial outer membrane protein, and an atypical GTPase that regulates mitochondrial transport along microtubules by linking mitochondria to kinesin and dynein motors (16). Degradation of Miro 1 is associated with arresting microtubule-based transport of damaged mitochondria to enable their efficient elimination (16). Analysis of the transcript levels of these proteins also showed a trend similar to that observed in Western blot analysis indicating a gradual decline during the course of infection (Fig. S3C).

### Dengue perturbs mitochondrial-selective autophagy (mitophagy).

**FIG 2:**
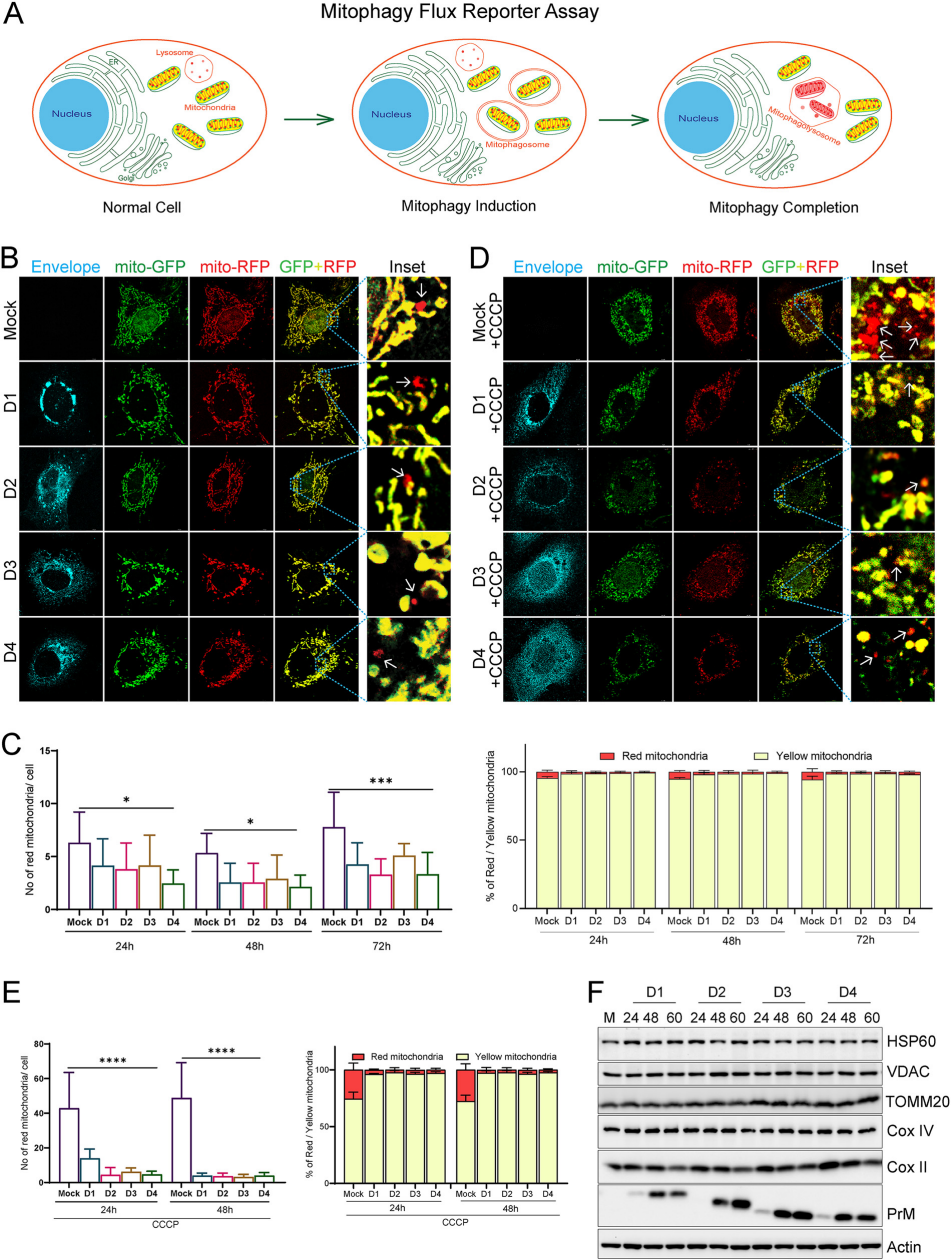
Dengue inhibits mitochondrial-specific autophagy (mitophagy). (A) Depiction of mitophagy reporter assay for the determination of mitophagy flux using the mitophagy reporter plasmid p-mito-mRFP-EGFP. The cells harboring the reporter display mitochondria with both EGFP (green) and mRFP (red) fluorescence under normal conditions. Upon mitophagy induction, the mitochondria are delivered to the lysosomes for degradation. In the acidic environment of the lysosomes mRFP is relatively stable, whereas GFP is rapidly degraded; hence, the mitochondria in the lysosomes only fluorescence red. (B) Huh7 cells were transfected with the mitophagy reporter plasmid and 16 h later were infected with the respective serotypes of dengue for the indicated time points. Dengue serotype-specific antienvelope antibody was used to stain the infected cells. Representative confocal images of mock or DENV-infected cells at 48 h postinfection. (C) Quantification of the average number of red mitochondria per cell for the indicated conditions and the percentage of red mitochondria with respect to the total mitochondria for the indicated conditions. (D) Representative confocal images of mock- and DENV-infected cells as in panel B but subjected to treatment with 20 μM CCCP for 12 h at 40 h postinfection to induce mitophagy. (E) Quantification of the average number of red mitochondria per cell and the percentage of red mitochondria with respect to the total mitochondria in cells subjected to CCCP treatment at 24 h and 48,h postinfection. For each quantitation about 20 to 25 cells were analyzed for the respective condition. (F) Western blot analysis of mock- and DENV-infected Huh7 cell to check the expression status of HSP60, VDAC, TOMM20, COXIV, and COXII at the indicated times post-dengue infection. Dengue PrM was used as the infection marker, and actin was used as the internal loading control. The data are the mean ± SEM of three independent experiments. Statistical analysis was determined using one-way ANOVA (C and E); *, *P < *0.05; ***, *P < *0.001; ****, *P < *0.0001.

To maintain cellular homeostasis, the cells attempt to resolve the mitochondrial stress and damage by promoting the rapid turnover of the damaged mitochondria. Selective autophagy of mitochondria, referred to as mitophagy, is an important aspect of mitochondrial quality control and rapid turnover of damaged mitochondria (5). Mitophagy leads to the engulfment of damaged mitochondria for subsequent lysosomal degradation (17). Since DENV inflicted severe mitochondrial injury in infected hepatic cells, we investigated the status of mitophagy in the DENV-infected cells. We used the mitophagy reporter p-mito-mRFP-EGFP (18) to evaluate the mitophagy flux. The mitophagy reporter works similarly to the autophagy traffic light reporter, taking advantage of the differential stability of the green fluorescent protein (GFP) and red fluorescent protein (RFP) in the acidic environment of the lysosomes (Fig. 2A) (19). Our observation suggests that DENV-infected cells did not show any signs of mitophagy (Fig. 2B), evidenced by the low number of red mitochondria per cell and the low percentage of red mitochondria with respect to the total mitochondria at various time points postinfection, which were also modestly lower than that observed in the respective mock-infected cells (Fig. 2C). To further confirm if dengue infection does not trigger mitophagy or if dengue infection leads to inhibition of mitophagy, we induced mitophagy by treatment with the mitochondrial uncoupler CCCP. Surprisingly, we observed that CCCP failed to induce mitophagy in Dengue-infected cells but induced mitophagy in mock-infected cells (Fig. 2D & E). To validate if the red mitochondria depict the mitochondria delivered to the lysosomal compartment, the mitophagy reporter transfected mock cells were subjected to CCCP treatment and immunostained with the lysosomal marker, Lamp1. We observed that all the red mitochondria puncta strongly colocalized with Lamp1, indicating that the mitochondria displaying only the red fluorescence are the ones delivered to the lysosomes for degradation (Fig. S4A). Determination of the colocalization between lysosome and mitochondria showed very minimal colocalization between the mitochondrial (TOMM20) and lysosomal (LAMP-1) markers in DENV-infected cells, in contrast to the mock-infected cells as evidenced by the colocalized spots observed between green and red channels (Fig. S4B). Treatment of DENV-infected cells with CCCP to induce mitophagy also did not lead to any significant increase in colocalization between TOMM20 and LAMP-1 (Fig. S4C). In correlation with this, we also did not observe an increase in colocalization between TOMM20 and LC3B in DENV-infected cells in comparison to mock-infected cells (Fig. S4D). Western blot analysis of the nuclear-encoded mitochondrial proteins TOMM20, VDAC, HSP60, and COX-IV did not reveal any significant decline in the expression status of these proteins; however, we observed reduced expression of the mitochondrion-encoded COX-II protein at a later time point postinfection (Fig. 2F and Fig. S5A). Treatment with bafilomycin A1, a vacuolar H+-ATPase inhibitor that blocks the acidification of lysosomes and prevents lysosomal degradation (20), also did not lead to the accumulation of these nuclear-encoded mitochondrial proteins, further indicating that the mitochondria do not undergo degradation in the lysosomes via autophagy (Fig. S5B). Evaluation of the formation of mitophagosome or engulfment of the damaged mitochondria in double-membrane vesicles (phagosomes) in DENV-infected cells through the proteinase K-based protease protection assay (Fig. S6A) showed that the DENV-infected cells lack mitophagosomes, as we did not observe any protection of the mitochondrial outer membrane (TOMM20), inner membrane (OPA1), and matrix (HSP60) proteins against proteinase K, whereas the CCCP-treated mock cell showed partial protection of these proteins against proteinase K (Fig. S6B). Overall, these observations suggest that mitophagy is not induced but is inhibited in DENV-infected hepatic cells, and the formation of mitophagosomes or mitochondrial engulfment in double-membrane vesicles is absent. Thereby, we observed the accumulation of damaged mitochondria during the course of infection due to a defect in their elimination via mitophagy in DENV-infected cells.

As we observed that dengue inhibits mitophagy, we wanted to determine which dengue protein(s) independently mediates this process. Huh7 cells were cotransfected with the plasmids expressing the respective dengue virus proteins in the pCMV-3tag-3a backbone and the mitophagy flux reporter (p-mito-mRFP-EGFP) to check their effect on the mitophagy flux. Quantification of the number of exclusively red mitochondria in the cells harboring the respective viral protein and the mitophagy reporter suggests that most of the viral proteins were exhibiting the same level of mitophagy flux (quantified by the average number of red mitochondria per cell) compared to the empty vector control (Fig. S7A and S7B); however, both NS4A- and NS4B-expressing cells display a negligible number of red mitochondria, which implicates these two dengue proteins in attenuation of mitophagy during dengue infection (Fig. S7A and S7B). Expression status of each individual protein was confirmed by Western blot analysis (Fig. S7C). DENV NS4B inhibits the mitochondrial fission protein DRP1 and promotes mitochondrial elongation (7). This may lead to inhibition of mitophagy, as the spatial segregation of the damaged mitochondria from the healthy mitochondrial reticulum is necessary for efficient mitophagy.

### Dengue promotes global autophagy.

**FIG 3:**
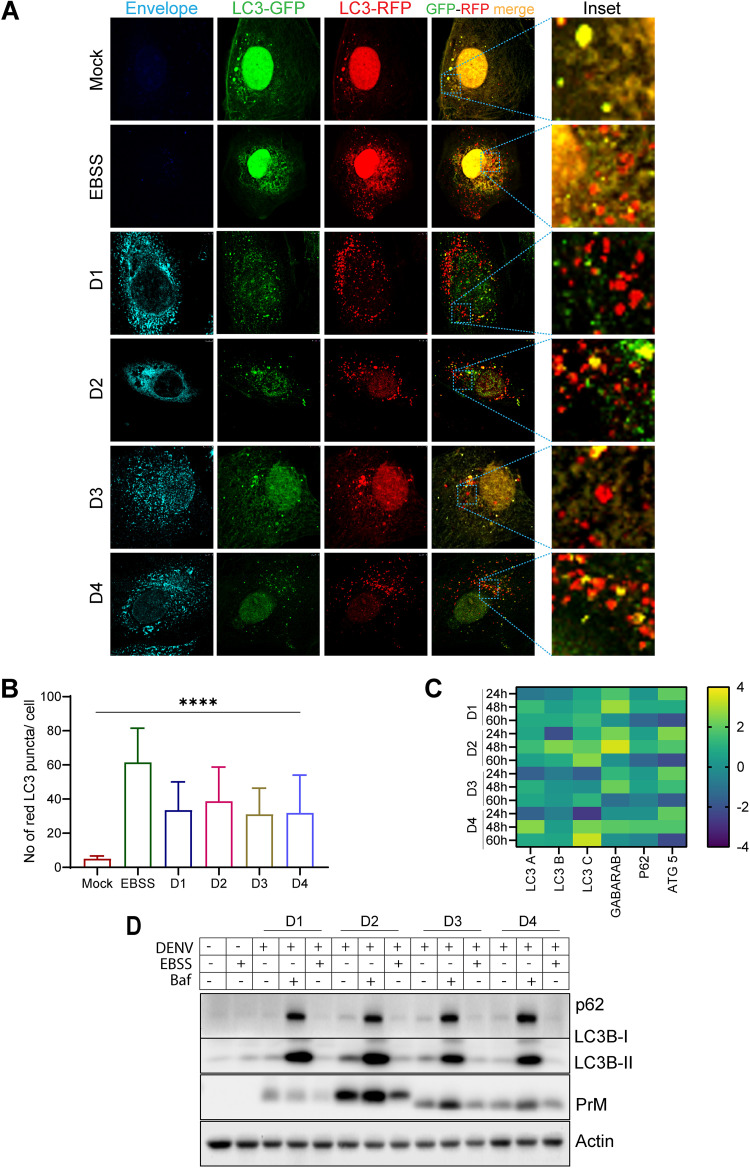
Dengue infection promotes global autophagy. To study autophagy, flux Huh7 cells were transfected with LC3 traffic light reporter plasmid p-mRFP-EGFP-LC3 and 16 h later were infected with the serotypes of dengue for 48 h. (A) Representative confocal images of mock-infected, EBSS-treated, and DENV-infected cells. The infected cells were stained with a serotype-specific dengue envelope antibody. (B) Quantification of the average number of red LC3 puncta per cell for the various serotypes at 48 h postinfection. About 20 cells were analyzed for each condition. (C) Heat map representing the transcript level of the indicated autophagy-specific genes in DENV-infected cells at the indicated time postinfection with respect to mock-infected cells. (D) Western blot analysis of p62 and LC3B I and II levels in mock- or DENV-infected Huh7 cells, either untreated or treated with EBSS (to induce autophagy) or bafilomycin (to suppress autophagy). PrM was used as an infection marker, and actin was used as an internal loading control. Postinfection (40 h), the cells were treated with EBSS for 1.5 h or bafilomycin-A for 8 h. Data are the mean ± SEM of three independent experiments. Statistical analysis was performed using one-way ANOVA. ****, *P < *0.0001.

In many instances, autophagy is induced during viral infection and can restrict viral infections by promoting their degradation, interferon production, and inflammatory response. However, many viruses usurp autophagic machinery to facilitate genome replication, virus release, and evasion of the innate immune response (21, 22). Many reports suggest that DENV promotes autophagy (23, 24) and also promotes lipid droplet-specific autophagy to promote lipid droplet processing and release of free fatty acids (25). As mitochondrial-selective autophagy was inhibited in DENV-infected cells, we evaluated the status of global autophagy, as an overall decline in global autophagy can also lead to inhibition of mitophagy. The autophagy flux assay using the traffic light reporter pTF-LC3-GFP-RFP (19) indicated that dengue triggers global autophagy flux (Fig. 3A and B) almost to the extent observed under nutrient-depleted conditions (Earle’s balanced salt solution [EBSS] treatment). The transcript levels of the majority of the autophagy genes tested, LC3A, LC3B, LC3C, GABARAB, p62, and ATG5, were highly upregulated by 48 hpi in DENV-infected Huh7 cells (Fig. 3C). Western blot analysis in DENV-infected cell lysates showed high levels of LC3B lipidation (LC3B-II) with respect to the mock control (Fig. 3D). LC3B lipidation is considered a primary marker of autophagy induction. We also observed a significant accumulation of the lipidated LC3B (LC3B-II) and p62 proteins in DENV-infected cells subjected to treatment with bafilomycin-A (Fig. 3D), confirming high autophagy flux during dengue infection.

### Dengue inhibits PINK1-PARKIN signaling.

**FIG 4:**
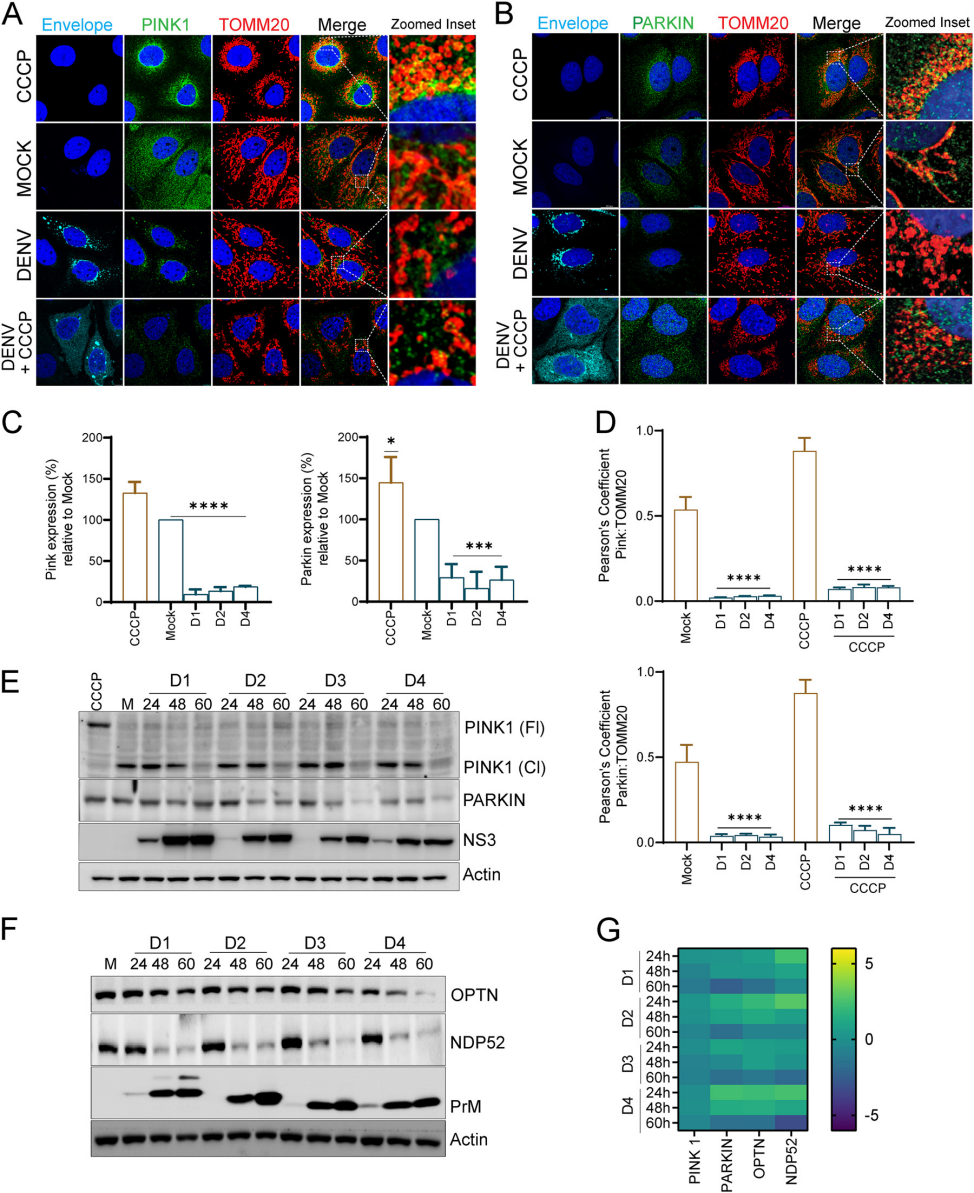
Dengue inhibits Parkin-mediated mitophagy. (A and B) Confocal images representing mock- or DENV1-infected Huh7 cells at 60 h postinfection were stained for infection (envelope), PINK1, and TOMM20 or stained for infection, Parkin, and TOMM20 as indicated. CCCP-treated Huh7 cells were used as a positive control for induction of mitophagy. Nuclei were counter stained with DAPI. The zoomed inset show regions of colocalization between the green and red channels as yellow spots. (C) Relative quantification of the expression of PINK1 and Parkin in the respective DENV-infected Huh7 cells in comparison to mock-infected and CCCP-treated Huh7 cells. (D) Bar graph depicting the Pearson coefficient of colocalization between PINK1/TOMM20 and Parkin/TOMM20 in the respective DENV-infected Huh7 cells in comparison to mock-infected and CCCP-treated Huh7 cells. (E) Western blot analysis of full length (Fl) and cleaved (Cl) PINK1 and Parkin levels in mock- or DENV-infected Huh7 cells at the indicated times postinfection. CCCP-treatment is used as the positive control for PINK1 activation. NS3 was used as the infection marker, and actin was used as the internal loading control. (F) Western blot analysis of NDP52 and optineurin in mock- or DENV-infected Huh7 cells at the indicated times postinfection. PrM was used as the infection marker, and actin was used as the internal loading control. (G) Heat map representing the transcript level of PINK1, Parkin, NDP52, and optineurin in DENV-infected cells at the indicated times postinfection with respective to mock-infected cells. Data are the mean ± SEM of three independent experiments. Statistical analysis was done by one-way ANOVA. *, *P < *0.05; **, *P < *0.01; ***, *P < *0.001 (E and G).

The PINK1-Parkin pathway which flags the damaged mitochondria for subsequent removal through autophagy is the most studied pathway of mitophagy (26). In healthy mitochondria, PINK1 is rapidly imported to the inner mitochondrial membrane (IMM) and degraded through the N-end rule pathway (27). When mitochondria are under stress, PINK1 import becomes defective, and it stabilizes on the outer mitochondrial membrane (OMM) (28). Active PINK1 promotes ubiquitin and Parkin phosphorylation, which triggers Parkin E3-ligase activity, resulting in ubiquitination of Parkin targets on the OMM (28). We investigated if DENV perturbs the PINK1-Parkin signaling to inhibit mitophagy. Confocal imaging of DENV-infected Huh7 cells 60 hpi demonstrated that both PINK1 and Parkin are downregulated in DENV-infected cells (Fig. 4A to C). Dengue infection also did not induce their colocalization to the mitochondria, evidenced by the low Pearson’s coefficient value obtained upon the merge of the green (PINK1/Parkin) and red (TOMM20) channels (Fig. 4D). When the mock- and DENV-infected cells were treated with CCCP to promote mitophagy, we observed that in mock-infected cells CCCP treatment led to the translocation of PINK1/Parkin to the mitochondria, resulting in enhanced colocalization between PINK1/Parkin and TOMM20, whereas, in DENV-infected cells we did not notice any significant increase in PINK1/Parkin translocation to the mitochondria (Fig. 4A, B, and D). Western blot analysis of PINK1 and Parkin in DENV-infected cells also confirmed that PINK1 and Parkin are downregulated in a time-dependent manner during the course of infection (Fig. 4E and Fig. S8A). Although we notice an accumulation of full-length PINK1 (PINK1-Fl) in CCCP-treated Huh7 cell lysates, we do not notice full-length PINK1 in DENV-infected cells (Fig. 4E and Fig. S8A). This suggests that despite mitochondrial stress, PINK1 stabilization does not occur in DENV-infected cells. PINK1 downregulation in DENV-infected cells was also evident by a gradual decline in the cleaved PINK1 (PINK1-Cl) during the course of infection (Fig. 4E and Fig. S8A). Western blots with purified subcellular fractions of mitochondria and cytosol also did not reveal mitochondrial stabilization/translocation of PINK1 and Parkin, which was evident in mitochondrial fractions of CCCP-treated Huh7 cells (Fig. S8B). Following Parkin-mediated ubiquitination of OMM proteins such as MFN2, VDAC, etc. the autophagy adaptors NDP52 and optineurin are recruited to the mitochondria, which then subsequently recruit the autophagy machinery to the mitochondria (28, 29). Knockdown of NDP52 and optineurin is reported to impede mitophagy (29, 30). Western blot analysis revealed that the expression status of both NDP52 and optineurin is down in DENV-infected cells, with the decline being more apparent in the later time points postinfection (Fig. 4F and Fig. S8A). In line with the Western blot data, we did observe downregulation of PINK1, Parkin, NDP52, and optineurin at the transcript level. Although we observed no change or a slight increase at the 24-h time point, we observed clear downregulation at the later time points postinfection (Fig. 4G). We also analyzed the expression status of some mitophagy adaptor proteins which function independently of Parkin by directly recruiting LC3B to the mitochondria, such as BNIP3, BNIP3L/Nix, and prohibitin. BNIP3 protein expression declined at the late time point, associated with an initial increase and decline in the transcript level. In contrast, BNIP3L/Nix showed a slight increase in the protein and transcript expression status with time postinfection (Fig. S9A and B). However, the expression of prohibitin was not affected at both the protein and transcript level during the course of infection (Fig. S9A and B). Irrespective of the expression status of the mitophagy adaptors, it is quite clear from the mitophagy flux assays that both the PINK1-Parkin-dependent and -independent mitophagy pathways are inhibited in DENV-infected hepatic cells (Fig. 2). Western blot analysis of bafilomycin-treated mock- and DENV-infected cells demonstrated that bafilomycin treatment did not lead to accumulation of Parkin but resulted in a slight accumulation of the 43-kDa cleaved fragment of PINK1, indicating that once released in cytosol, it is further degraded via the autophagy process (Fig. S9C). However, we observed a good accumulation of NDP52 and optineurin (Fig. S9D). The lack of accumulation of full-length PINK1 and Parkin indicate that they are not consumed via mitophagy or other processes involving lysosomal degradation. The accumulation of NDP52 and optineurin despite the inhibition in mitophagy suggests that these conventional autophagy adaptors are functionally involved in other mechanisms associated with autophagy in mock- and DENV-infected cells.

### Dengue inhibits mitochondrial biogenesis.

**FIG 5:**
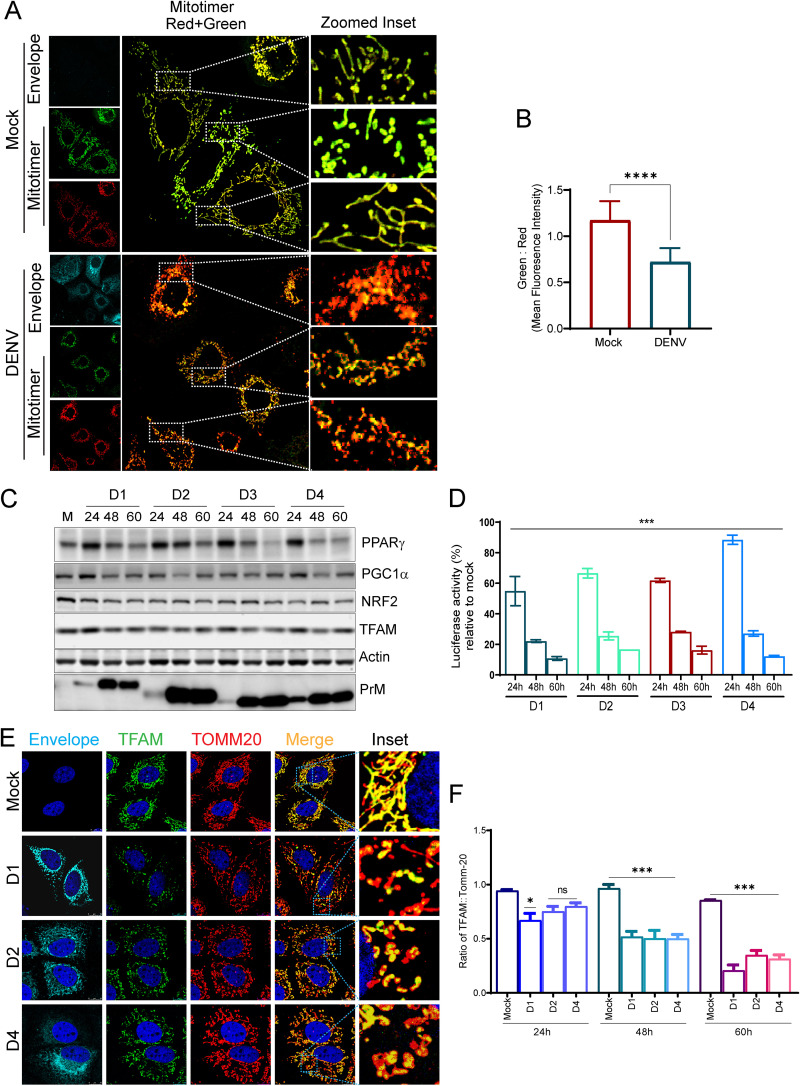
Dengue inhibits mitochondrial biogenesis in Huh7 cells. (A) The mito-timer reporter plasmid harboring the fluorescence timer DsRed1-E5 tagged to the mitochondrial localization sequence was used to determine the status of mitochondrial biogenesis during dengue infection. The mito-timer reporter was transfected in Huh7 cells, and 16 h later the cells were mock infected or infected with dengue. Confocal images representing mock- or DENV1-infected Huh7 cells 48 h postinfection. (B) Ratiometric analysis of relative mean fluorescence intensity of green:red fluorescence in mock- and DENV 1-infected cells. (C) Western blot analysis for relative expression of PPARγ, PGC1α, NRF2, and TFAM in mock- and DENV-infected cells at the indicated times postinfection. PrM was used as an infection marker, and actin was used as the internal loading control. (D) PGC1α promoter activity in mock- and DENV-infected Huh7 cells. The column graph depicts renilla luciferase-normalized firefly luciferase activity in Huh7 cells cotransfected with PGC1α-promoter-based firefly luciferase and HSV-thymidine kinase promoter renilla luciferase reporter for 16 h followed by infection with the respective dengue serotypes for the indicated time points postinfection. The PGC1α promoter activity is expressed as a percentage of the normalized reporter activity in mock-infected cells. (E) Confocal images of mock- and DENV-infected cells were fixed at 60 h postinfection and stained for infection (envelope), TFAM (green), and TOMM20 (red). (F) Column graph depicting the ratiometric analysis of relative mean fluorescence intensity of TFAM (green):TOMM20 (red) in mock- and DENV-infected cells at the indicated time points postinfection. The data represent the mean ± SEM of three independent experiments. Statistical analysis was performed using Student’s *t* test (B) or one-way ANOVA (D and F). ns, nonsignificant; *, *P < *0.05; ***, *P < *0.001; ****, *P < *0.0001.

Mitophagy and mitochondrial biogenesis work in coordination to facilitate the rapid turnover of damaged mitochondria and maintenance of mitochondrial homeostasis (17). Many viruses have been shown to affect the host mitochondrial biogenesis (31). We investigated the status of mitochondrial biogenesis during dengue infection. We used a mito-timer reporter harboring the fluorescence timer DsRed1-E5, which is a mutant of the DsRed and displays a green to red shift in fluorescence as the protein matures and can be used to track mitochondrial biogenesis (32). We observed that the mito-timer localized to the mitochondria in both mock- and DENV-infected cells; however, only the DENV-infected cells primarily displayed intense red fluorescent mitochondria 48 h postinfection with minimal green fluorescence, whereas the mock-infected cells displayed both red and green fluorescent mitochondria of almost similar intensity (Fig. 5A). Ratiometric analysis of mito-timer green:red fluorescence revealed higher red fluorescence in DENV-infected cells, suggestive of lower mitochondrial turnover (Fig. 5B). The human mitochondrial genome possesses 37 genes including the 13 proteins involved in oxidative phosphorylation; however, >1,000 genes are encoded by the nuclear genome, which is controlled by a network of transcriptional factors, coactivators, and corepressors (33, 34). Peroxisome proliferator-activated receptor (PPAR)γ and its coactivator PGC1α serve a wide range of biological functions, including mitochondrial biogenesis and turnover (33, 34). We observed that PPARγ and PGC1α are downregulated during the course of dengue infection (Fig. 5C and Fig. S10A). Assessment of PGC1α promoter activity (35) also confirmed downregulation of PGC1α transcriptional activity during dengue infection (Fig. 5D). PGC1α works in tandem with NRF2 and coactivates NRF1, and subsequently the NRFs activate the transcription of nuclear-encoded mitochondrial proteins, including the mitochondrial transcription factor A (TFAM), which plays a crucial role in mitochondrial gene transcription and maintenance of mitochondrial genome copy number (32). We observe that dengue downregulated NRF2 expression during the course of infection with a concomitant downregulation in the expression of TFAM (Fig. 5C and Fig. S10A). Downregulation of PGC1α and NRF2 was also evident at the transcript level (Fig. S10B). Confocal microscopy of dengue-infected cells further validated the gradual decline in TFAM levels resulting in a decline in the TFAM:TOMM20 ratio during the course of infection (Fig. 5E and F). Taken together, these observations suggest the downregulation of mitochondrial biogenesis during dengue infection.

### Dengue infection leads to necrotic cell death in hepatic cells.

**FIG 6:**
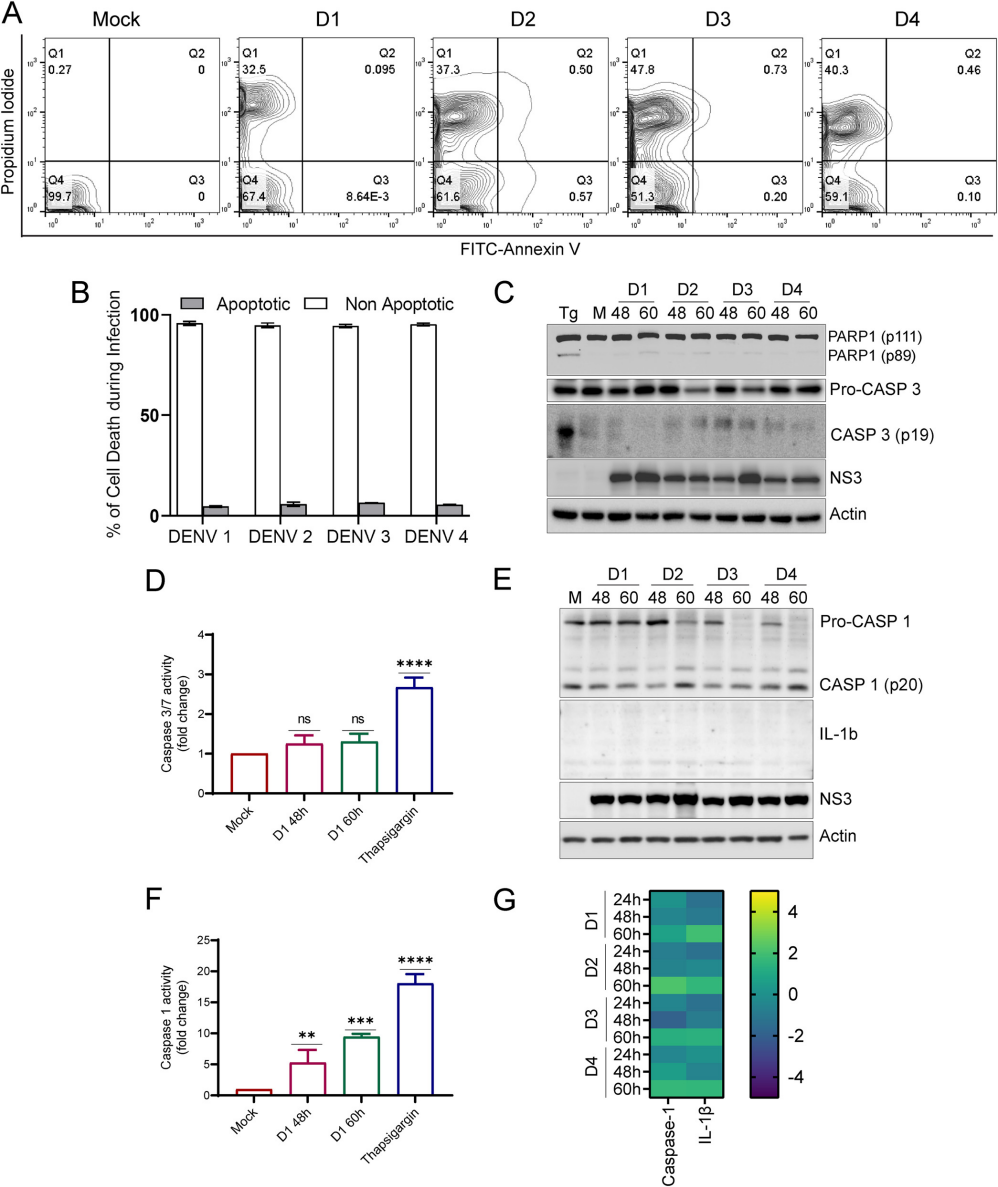
Dengue infection leads to necrotic cell death in Huh7 cells. (A) Cell death analysis by FACS in mock- and DENV-infected cells at 60 h postinfection. Contour plot showing FACS analysis of mock-infected and infected Huh 7 cells with the *x y* axes representing annexin V and PI staining, respectively. (B) Bar graph depicting the percentage of cells undergoing apoptotic and nonapoptotic cell death in Huh7 cells infected for 60 h with various serotypes of dengue. (C) Western blot analysis of PARP1 cleavage and caspase 3 activation in mock- and DENV-infected Huh7 cells at the indicated times postinfection. NS3 was used as the infection marker, and actin was used as the internal loading control. (D) Bar graph depicting caspase 3/7 activity in mock- and DENV-infected cells at the indicated times postinfection. The activity is plotted as the fold change with respect to mock-infected cells. (E) Western blot analysis of caspase 1 activation and IL-1β in mock- and DENV-infected Huh7 cells at the indicated times postinfection. NS3 was used as the infection marker, and actin was used as the internal loading control. (F) Bar graph depicting caspase 1 activity in mock- and DENV-infected cells at the indicated times postinfection and represented as the fold change with respect to the mock group. (G) Heat map representing the transcript levels of caspase 1 and IL-1β in infected Huh7 cells with respect to mock-infected cells. The data presented are the mean ± SEM of three independent experiments. Statistical analysis was done by one-way ANOVA. ns, nonsignificant; **, *P < *0.01; ***, *P < *0.001; ****, *P < *0.0001.

The lack of mitochondrial homeostasis and quality control in DENV-infected Huh7 cells may compromise cellular homeostasis and lead to cellular injury and necrosis. We did a flow cytometry assay combining the staining with annexin V and propidium iodide (PI) to discriminate between early apoptotic, late apoptotic, and necrotic cell death. DENV-infected Huh7 cells at 60 hpi were found to be more necrotic as they were highly stained with PI, which indicates loss of membrane integrity, an early sign of necrosis (Fig. 6A). In contrast, minimal cells showed annexin V positivity, a common sign of early apoptotic cells due to the translocation of phosphatidylserine from the inner to the outer layer of the plasma membrane (Fig. 6A). The estimation of the proportion of cells undergoing necrosis versus apoptosis indicated that the majority of DENV-infected Huh7 cells undergo necrosis (Fig. 6B). Further, we performed Western blot analysis for apoptotic markers, such as caspase 3 and PARP-1. Caspase 3 is the primary executioner caspase in apoptosis and is responsible for the proteolytic cleavage of different proteins, including PARP-1, which is involved in DNA repair (36). In comparison to cell lysates obtained from thapsigargin (Tg)-treated Huh7 cells, we did not observe the proteolytically cleaved fragment of caspase 3 (p-19 fragment), which represents the active caspase, but we did notice a slight increase in the cleaved form of PARP-1 at a later time point postinfection (Fig. 6C and Fig. S10C). In line with these observations, the caspase 3/7 activity assay also indicated only a slight but nonsignificant increase in the activity at 48 h and 60 h postinfection with DENV-1, in contrast to the sharp increase observed in thapsigargin-treated mock cells (Fig. 6D). Defects in quality control and the elimination of damaged mitochondria can lead to leakage of damage-associated molecular patterns (DAMPs) and trigger the NLRP3 inflammasome pathway (37). When activated, these multimeric signaling complexes or inflammasomes lead to the activation of the inflammatory cysteine protease caspase-1, which then facilitates the processing of the proinflammatory cytokines such as interleukin-1β (IL1β) and IL-6 (37). Western blot analysis of DENV-infected Huh7 cells showed a decrease in the procaspase 1 levels at the later time point postinfection with a concomitant increase in the cleaved/active form of caspase-1 (p20) (Fig. 6E and Fig. S10C). However, we failed to detect both the pro and active form of IL-1β in these lysates (Fig. 6E). Caspase-1 activity assay also showed a significant increase in DENV-infected Huh7 cells at 48 and 60 hpi compared to the mock cells (Fig. 6F). The transcript levels of caspase-1 and IL-1β were also found to be upregulated at later time points post-dengue infection in Huh7 cells (Fig. 6G). Huh7 represents liver epithelial cells and may not represent a good model to study inflammasome signaling, which may be the reason that we failed to detect IL-1β and other players involved in inflammasome signaling and pyroptosis. Overall, these observations support the notion that DENV infection of hepatic cells promotes mitochondrial and cellular injury triggering necrotic cell death.

### Dengue infection leads to the release of mt-DAMPS.

**FIG 7:**
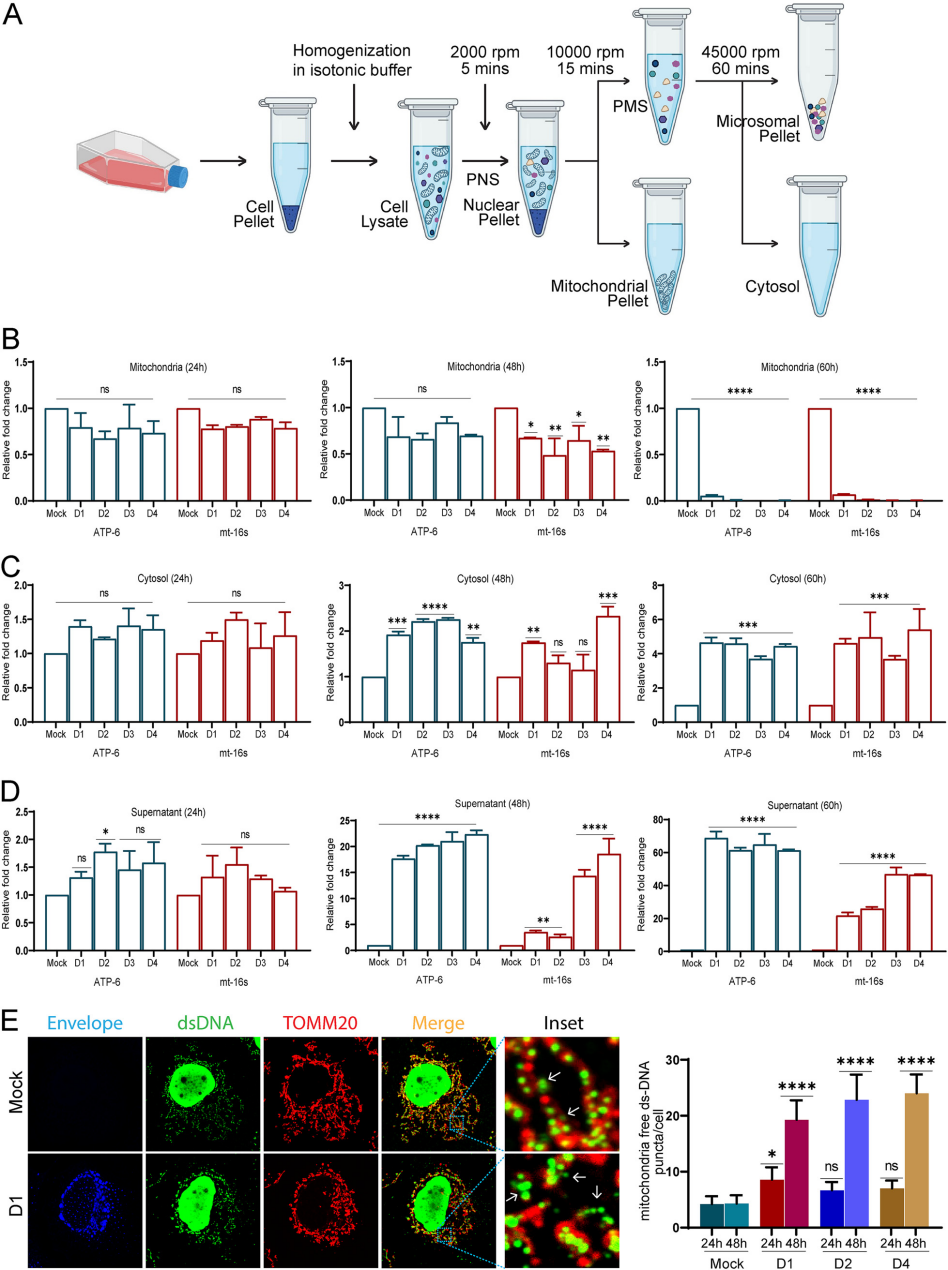
Dengue virus infection leads to mt-DNA release in Huh7 cells. (A) Work flow illustrating the isolation of mitochondrial and cytosol subcellular fractions through differential centrifugation. (B) mt-DNA isolated from the mitochondrial fractions was subjected to quantitative real-time PCR (qRT-PCR) for mitochondrial ATP-6 and mt-16S RNA. Bar graph depicting their relative levels (fold change with respect to mock infection group) in the mitochondrial fractions obtained from mock- and DENV-infected cells at the indicated times postinfection. (C) mt-DNA isolated from the cytosol fractions of the indicated samples was subjected to qRT-PCR. Bar graph depicting the relative levels (fold change with respect to mock infection group) of ATP6 and mt-16S RNA. (D) mt-DNA isolated from the preclarified cell culture medium of the indicated samples was subjected to qRT-PCR. Bar graph depicting the relative levels (fold change with respect to mock) of ATP6 and mt-16S RNA. (E) Confocal images of mock- and DENV serotype 1 (D1)-infected Huh7 cells 48 h postinfection stained for infection (envelope), dsDNA (green), and TOMM20 (red). The bar graph on the right depicts the quantitation of mitochondrial free dsDNA puncta found in the cytosol of mock-infected and infected cells at 24 h and 48 h postinfection with different serotypes of dengue. Data are the mean ± SEM of three independent experiments. Statistical significance was determined using one-way ANOVA. ns, nonsignificant; *, *P < *0.05; **, *P < *0.01; ***, *P < *0.001; ****, *P < *0.0001.

Mitochondrial stress causes mitochondrial depolarization and opening of the permeability transition pore (mPTP), which can result in the leakage of mitochondrial contents into the cytoplasm. Our previous results demonstrate that dengue infection leads to the opening of the mPTP (Fig. 1G). VDAC oligomerization on the OMM can also trigger mPTP opening and release of mt-DNA into the cytoplasm (38). To determine mt-DNA leakage or release from the mitochondria during dengue infection, we prepared subcellular fractions of DENV-infected Huh7 cells through differential centrifugation to obtain enriched fractions of mitochondria and pure cytosol (Fig. 7A). The mt-DNA from the mitochondrial fraction, pure cytosol, and culture supernatants was isolated and subjected to quantitative real-time PCR (qRT-PCR) using primers specific for the mitochondrial 16S rRNA and ATP6 genes. We observed substantial depletion in the mt-DNA from the mitochondrial fractions of DENV-infected cells at 60 h postinfection and a slight but nonsignificant decline at 24 h and a modest decline at 48 hpi (Fig. 7B). During the course of infection, we observed a concomitant increase in the levels of mt-DNA in the cytosol at 48 hpi (~2-fold) and 60 hpi (~4- to 6-fold) (Fig. 7C). Interestingly, we observed a rise in the levels of mt-DNA in the culture supernatants of infected cells from 24 hpi with a steep increase of ~15- to 20-fold at 48 hpi and ~20- to 50-fold at 60 hpi (Fig. 7D). Mitochondrial DNA is a small circular DNA molecule packaged into nucleoprotein complexes called nucleoids. TFAM, the mitochondrial transcription factor A, binds to mt-DNA in the mitochondrial matrix and controls its architecture, packaging, abundance, and segregation (39). Deficiency in TFAM is implicated in mitochondrial stress and mis-packaged mt-DNA that is eventually ejected into the cytoplasm (40). To investigate the mt-DNA architecture, the DENV-infected cells were immunostained with TOMM20, and double-stranded DNA (dsDNA) antibody to label the mitochondria and mt-DNA (Fig. 7E). The mock-infected cells displayed proper mt-DNA nucleoids evenly dispersed within the mitochondria, whereas the DENV-infected cells displayed unevenly dispersed irregular mt-DNA nucleoids, with a good number of nucleoids devoid of TOMMM20 staining around them (Fig. 7E). Quantification of the mt-DNA puncta falling outside the mitochondrial periphery clearly showed a significant rise in their number in DENV-infected cells at 48 hpi in comparison to mock-infected cells (Fig. 7E). Similar to Huh7 cells, when A549 cells and HEK cells were infected with DENV-2, we observed that there was an exponential increase in mt-DNA levels at 48 and 60 hpi in the cytosol and culture supernatant of both the cell lines (Fig. S11A to D). Overall, these observations substantiate that dengue infection of liver cells is associated with profound levels of mitochondrial injury and stress, leading to massive release/leakage of mt-DNA into the cytoplasm and extracellular milieu.

**FIG 8:**
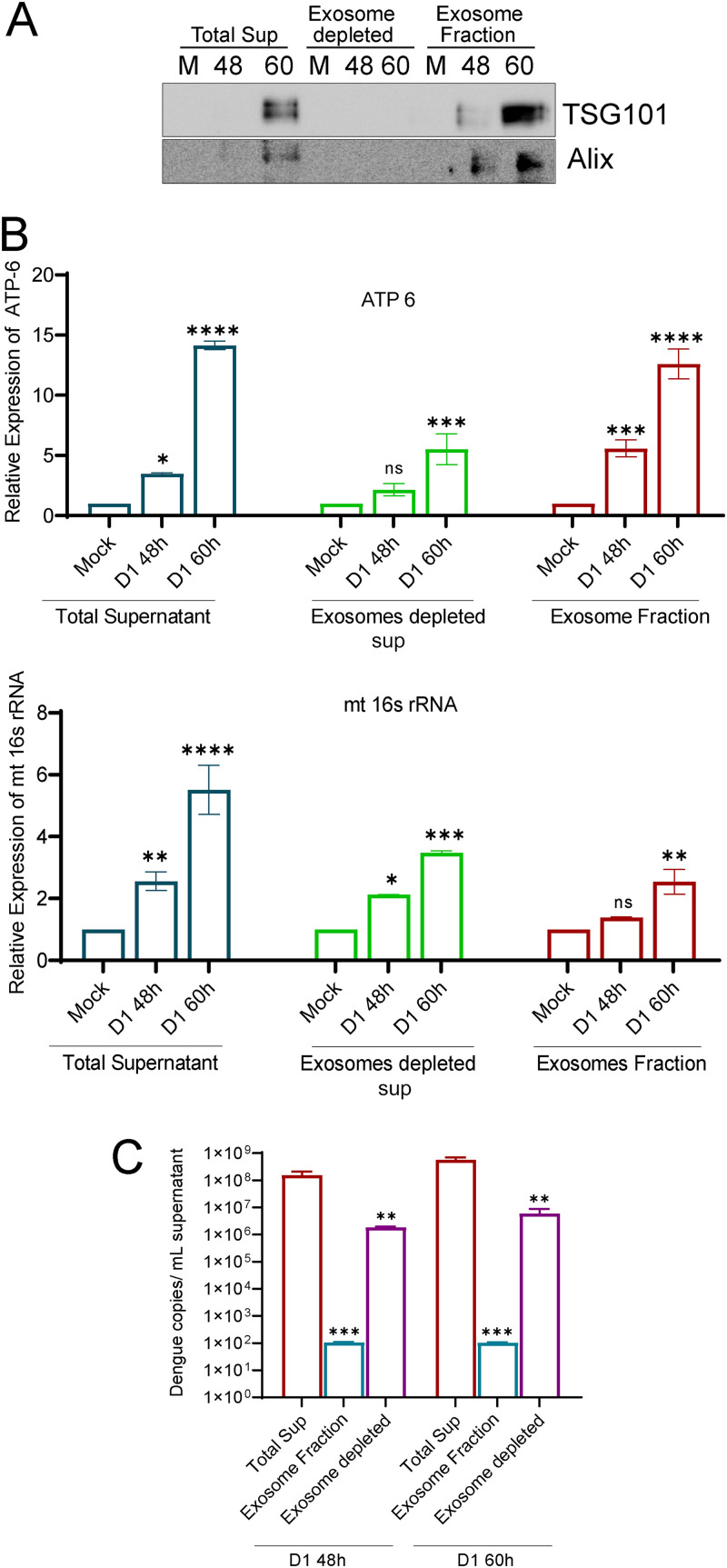
mt-DNA release through exosomes. Culture supernatants from mock- and dengue serotype 1-infected cells at 48 h and 60 h postinfection were used to isolate exosomes. (A) Western blot analysis of the exosomes and exosome-depleted fractions with TSG101 and Alix to determine the purity of exosomes. Mt-DNA was isolated from the exosome fraction, its respective exosome depleted culture supernatant, and the culture supernatant equal in volume to that used for exosome isolation. qRT-PCR was done for ATP6 and the mt-16S RNA gene. (B) Bar graph depicting the relative expression of ATP6 and mt-16S rRNA in exosomes and exosome-depleted and total supernatant. (C) Estimation of Dengue copy number in the unfractionated culture supernatant, exosome fraction, and exosome-depleted culture supernatant. Data are the mean ± SEM of three independent experiments. Statistical significance was determined using one-way (ANOVA). ns, nonsignificant; *, *P < *0.05; **, *P < *0.01; ***, *P < *0.001; ****, *P < *0.0001.

Since we observed a significant release of mt-DNA into the cell culture supernatants as early as 24 and 48 hpi, which is long before the DENV-induced necrotic cell death is triggered, we investigated if mt-DNA is released from DENV-infected cells in an active mode through the exosome. The membrane-bound extracellular vesicles or exosomes facilitate cell-to-cell communication and possess the capacity to trigger inflammation. Many studies have shown that the exosome contains DNA, including mitochondrial DNA, RNA, mRNA, microRNAs, and long noncoding RNAs (41). To test the release of mt-DNA through exosomes, we purified exosomes from the culture supernatant collected from mock- or DENV1-infected Huh7 cells at 48 hpi. The total culture supernatant, exosome-depleted supernatant, and purified exosome fraction were subjected to Western blot analysis with TSG101 and Alix, two well-known markers for exosomes (42), to verify the enrichment of exosomes (Fig. 8A). The exosome fraction, the respective exosome-depleted fraction, and the unfractionated culture supernatants (volume similar to the volume used for exosome isolation) were subjected to mt-DNA purification and qRT-PCR for mitochondrial genes. We used exosome-depleted fetal bovine serum (FBS) throughout this experiment to avoid contamination with the exosome from the FBS source. We observed an increase in the levels of mt16S rRNA and ATP6 at 48 and 60 hpi in both the exosome fraction and exosome-depleted fraction (Fig. 8B). However, the increase observed from 48 to 60 h in the exosome-depleted fraction was modestly higher than that of the exosomal fraction (Fig. 8B). Based on these observations, it is evident that the release of cell-free mt-DNA into the extracellular milieu during dengue infection is a consequence of release through exosome-dependent and -independent mechanisms and during the late time points may be an outcome of cell death through necrosis. Examination of dengue RNA in these fractions suggested that the exosome fraction has a lower level of DENV RNA than the exosome-depleted culture supernatant (Fig. 8C), suggesting that the DENV virus particles and/or genome may also be present in secreted exosomes, but much less than to the levels observed in exosome-free supernatant.

### Supernatants from DENV-infected hepatic cells trigger a proinflammatory response in challenged naive monocytes and macrophages.

**FIG 9:**
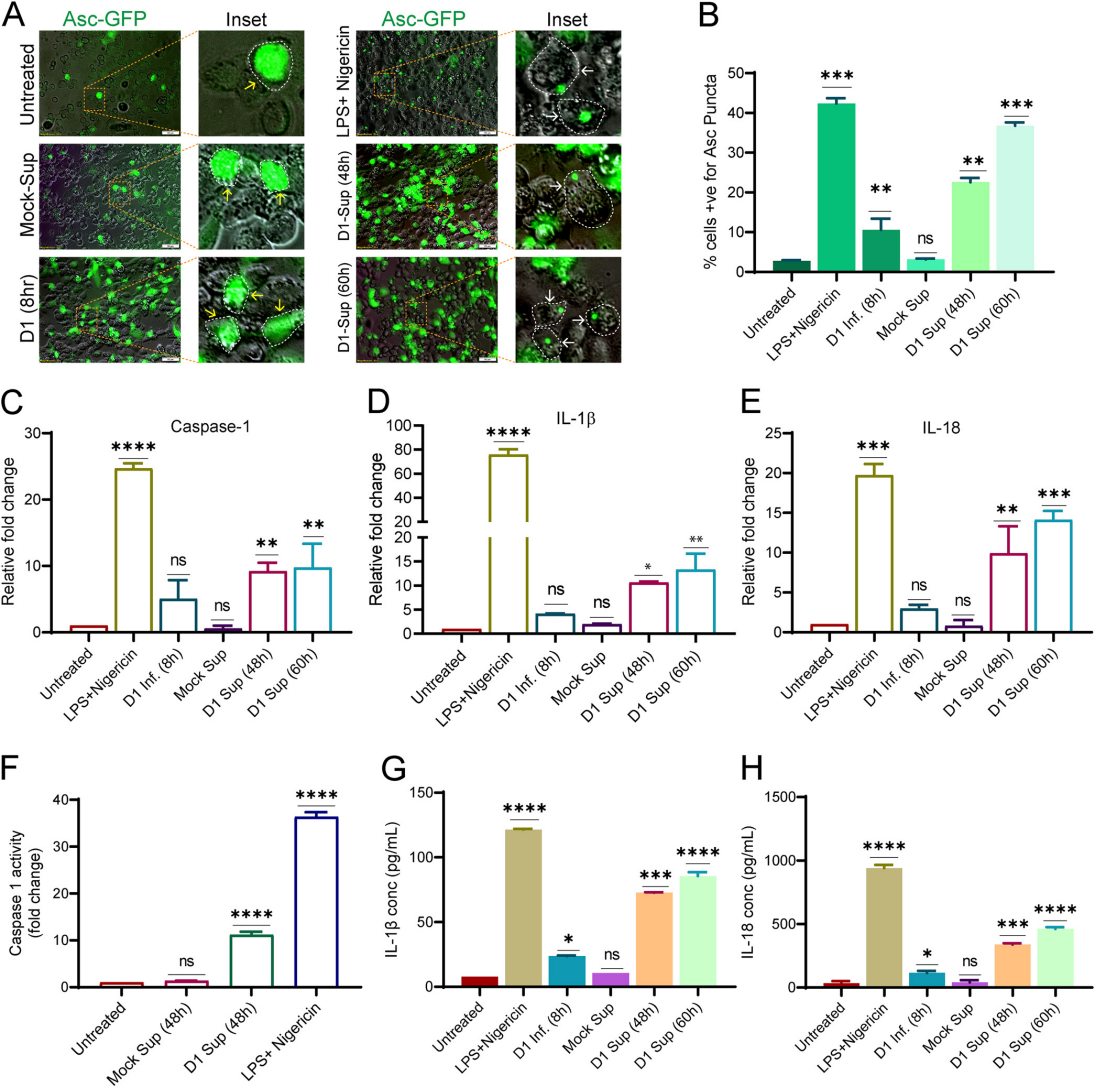
Dengue infection releases mt-DAMPs, which leads to inflammasome activation in monocytes and macrophages. (A) The inflammasome activation reporter monocyte cell line THP-ASC-GFP-derived macrophages, were challenged for 8 h with preclarified culture supernatants obtained from DENV-infected (serotype 1)-Huh7 cells at 48 h and 60 h postinfection. Challenge with culture supernatant from mock-infected Huh7 cells, LPS+Nigericin, and direct infection with dengue virus 1 was used as the negative, positive, and infection control. The upregulation of ASC-GFP expression (inflammasome priming) and ASC-GFP specks formation is represented by yellow and white arrows in the respective zoomed insets. The cell periphery is indicated by dotted lines. (B) Bar graph depicting the quantification of the number of ASC-GFP puncta/specks per cell 8 h postchallenge with the respective treatments. (C to E) Graphs depicting the transcript levels of caspase 1 (C), IL-1β (D), and IL-18 (E), in THP1 monocytes challenged for 8 h with the respective treatments described in panel A. The data are presented as the fold change with respect to untreated THP1 monocytes. (F) Graph depicting the relative caspase 1 activity in THP1 monocytes 8 h postchallenge with the indicated treatments. (G and H) Graph depicting the relative quantity of IL-1β and IL-18 released into the culture supernatants from the activated THP1 monocytes 8 h postchallenge with the treatments indicated in panel A. Data are the mean ± SEM from three independent experiments. Statistical significance was determined using one-way ANOVA. ns, nonsignificant; *, *P < *0.05; **, *P < *0.01; ***, *P < *0.001, ****, *P < *0.0001.

mt-DNA is a potent danger signal which is recognized by the innate immune system, leading to activation of proinflammatory signaling. Hence, we tested if the challenge of naive THP1 monocytes with the culture supernatants from DENV-infected hepatic cells is enough to promote proinflammatory signaling. The canonical inflammasomes are made of multimeric protein complexes that sense the pathogen-associated molecular patterns (PAMPs) or DAMPs such as mt-DNA (43). Upon activation, the receptor oligomerizes and binds to the adaptor protein ASC through the PYD domains, and ASC binds with procaspase-1 through its CARD domain to form the complete structure (43). Two signals govern the inflammasome signaling; the first priming signal involves the activation of the pattern recognition receptors (PRRs) leading to the upregulation of the inflammasome components and cytokines. The second induction signal leads to the assembly and activation of the complex, resulting in the activation of caspase 1 and maturation of the pro-interleukin-1β and -18 (43). We used the ASC speck reporter monocyte (THP-ASC-GFP)-derived macrophages for this purpose. At the resting stage, these cells display weak or no GFP expression; upon priming the GFP fluorescence signal increases and is visible throughout the cytoplasm, whereas upon inflammasome activation, ASC-GFP polymerization into a speck-like architecture is evident as bright GFP puncta/dots (Fig. 9A; compare untreated with supernatants obtained from mock-infected cells (mock-sup) and LPS+Nigericin treated). THP-ASC-GFP monocyte-derived macrophages treated for ~8 h with supernatant from the DENV-infected cells displayed a higher percentage of cells with ASC specks than the mock-sup treated or infected cells at 8 hpi (Fig. 9A). The culture supernatant obtained 60 h postinfection had a better effect than the 48-h culture supernatant, almost to the extent observed in the cells treated with lipopolysaccharide (LPS) and nigericin (Fig. 9A and B). Analysis of the transcript levels after the challenge of THP1 monocytes with the respective cell culture supernatants from DENV-infected Huh7 cells showed a significant upregulation of caspase 1, IL-1β, and IL-18 (Fig. 9C to E), in agreement with the observations made with THP-ASC-GFP reporter monocytes. The caspase 1 activity assay also confirmed that caspase-1 is activated after the challenge of THP1 monocytes with supernatant from DENV-Huh7 cell (Fig. 9F). The release of IL-1β and IL-18 from the activated THP1 monocytes was also confirmed by enzyme-linked immunosorbent assay (ELISA), which further validated the activation of the inflammasome and proinflammatory signaling in these cells (Fig. 9G and H). In line with this evidence, the challenge of human peripheral blood mononuclear cells (PBMCs) with culture supernatants from DENV-infected Huh7 cells also led to the transcriptional upregulation of IL-1β and IL-18 and caspase 1 (Fig. S12A), further confirming the proinflammatory potential of the culture supernatants from DENV-infected hepatic cells. To determine if the inflammasome activation upon treatment with culture supernatants from DENV-infected cells was primarily due to the presence of mt-DNA, we pretreated the culture supernatant with DNase and then challenged the naive THP1 monocytes. Our observation suggests that treatment with DNase resulted in a marked decline in the inflammasome activation, evidenced by a decline in the upregulation of IL-1β, IL-18, and caspase-1 compared to untreated culture supernatants (Fig. S12B). This suggests that mt-DNA in the culture supernatants obtained from DENV-infected cells is the primary trigger of inflammasome. We also observed that the exosome fraction and exosome-depleted fraction obtained from the culture supernatants of DENV-infected cells were equally capable of activating the inflammasome, as both were able to induce ASC speck formation in THP-ASC-GFP monocyte-derived macrophages (Fig. S12C).

### Dengue patient sera have high loads of cell-free mt-DNA.

**FIG 10:**
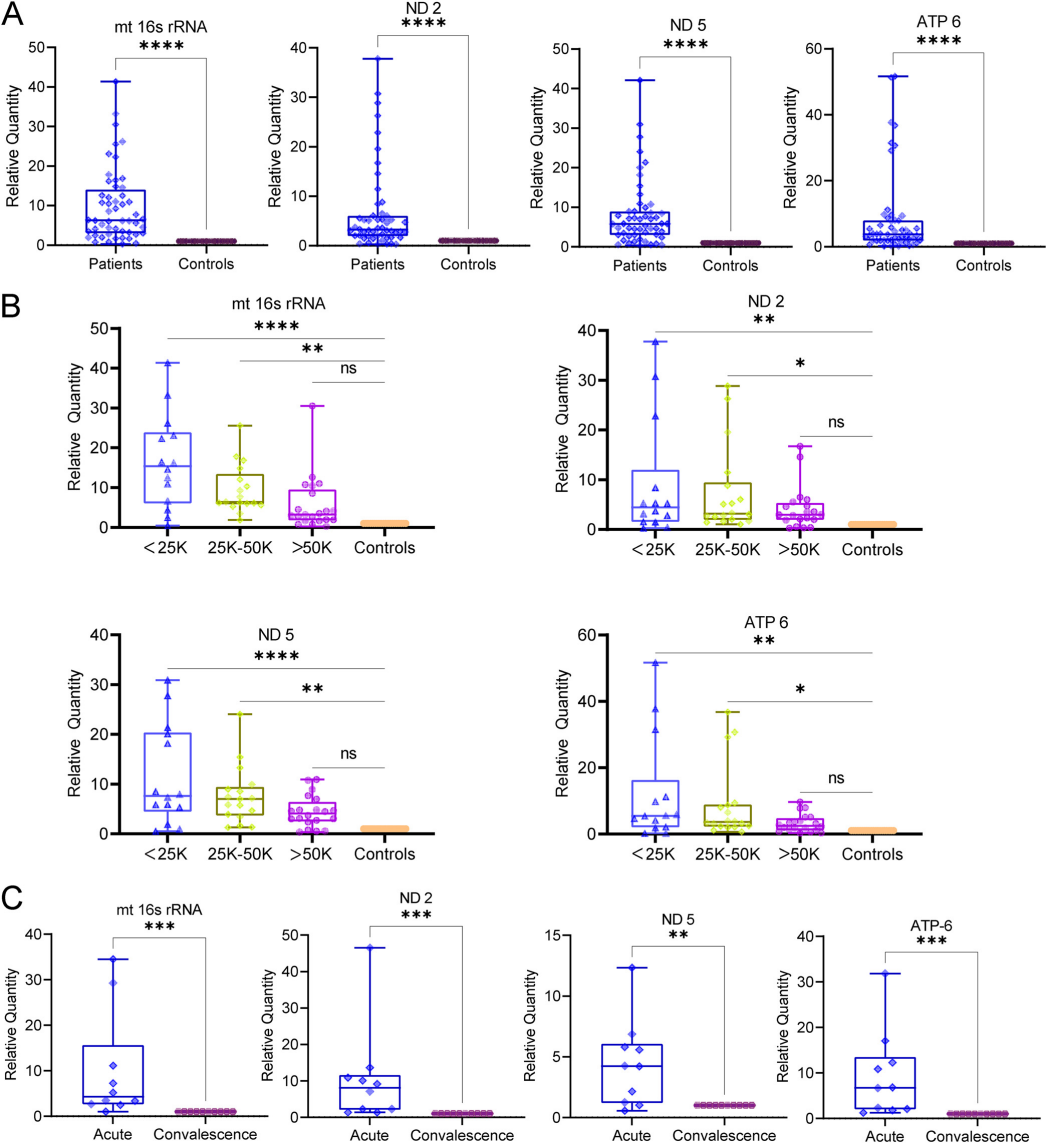
Cell free mt-DNA in the plasma samples of Dengue-infected patients. Peripheral blood samples were collected from laboratory-confirmed dengue patients and age-matched healthy controls. mt-DNA was isolated from the plasma and subjected to qRT-PCR for the mitochondrial encoded genes, mt-16s rRNA, ND2, ND5, and ATP 6. (A) Graph depicting the relative quantity of the indicated genes encoded from mitochondrial genome in the plasma samples of dengue patients (*n* = 53) and healthy controls (*n* = 20). (B) Graphs depicting the relative quantity of the indicated mitochondrial genes in dengue patient plasma samples segregated on the basis of decline in platelet count in comparison to healthy controls (*n*; <25,000 = 15, 25,000 to 50,000 = 18, >50,000 to 75,000 = 20, healthy control = 20). (C) Graphs depicting the relative quantity of the indicated mitochondrial genes in paired plasma samples obtained from dengue patients during the acute and convalescent phases of infection (*n* = 10). Statistical significance was determined using Student’s *t* test (A and C) or one-way ANOVA (B). ns, nonsignificant; *, *P < *0.05; **, *P < *0.01; ***, *P < *0.001; ****, *P < *0.0001.

A wide range of human diseases, including trauma, acute myocardial infarction, acetaminophen-induced acute liver injury, community-acquired bacterial meningitis, and sepsis, have been found to be associated with high loads of cell-free mt-DNA in the circulation (44). Our *in vitro* observations establish that the leakage or release of mitochondrial DAMPs such as mt-DNA into the extracellular milieu from infected hepatic cells can trigger proinflammatory signaling in human monocytes, monocyte-derived macrophages, and PBMCs (Fig. 9). Hence, we investigated the status of cell-free mt-DNA in the blood samples of dengue patients. Peripheral blood was collected from laboratory-confirmed dengue patients and their age-matched healthy controls. Serum samples were used for the isolation of cell-free DNA using a blood DNA isolation kit, and mt-DNA levels were estimated by quantitative PCR (qPCR) using gene-specific primers against mt16s rRNA, ND2, ND5, and ATP6. We targeted genes spanning the entire mitochondrial genome to determine whether mt-DNA is released in random (either in its entirety or fragments) or only specific regions of mt-DNA. We segregated the samples into three groups; (i) comparison between dengue patients and healthy controls, (ii) comparison between the samples on the degree of thrombocytopenia, and (iii) comparison in the same individuals at the acute and convalescent stages of the disease. Our findings reveal that all four mitochondrial genes (mt16s rRNA, ND2, ND5, and ATP-6) were highly elevated (~40 to 50 times) in dengue-infected patients compared to healthy controls (Fig. 10A). We also analyzed the data based on platelet count and grouped them as those with platelets of less than 25,000, 25,000 to 50,000, and higher than 50,000. Interestingly, we observed that the level of cell-free mt-DNA strongly correlated with the degree of thrombocytopenia, with the mt-DNA levels decreasing with an increase in platelet count (Fig. 10B). The degree of thrombocytopenia is a major indicator of progression to severe dengue; hence, these observations indicate that cell-free mt-DNA may play an important role in driving progression toward severe dengue disease. A major drawback of the human samples is the high degree of heterogeneity within the cohort; hence, we analyzed the mt-DNA within individuals as they progressed from the acute (1 to 7 days post-onset of fever) to convalescent (past 7 days post-onset of fever) phases of dengue infection. We observed that the mt-DNA levels were severalfold higher in the acute phase than in the convalescent phase (Fig. 10C). High ROS from damaged mitochondria can lead to the oxidation of mt-DNA, and oxidized mt-DNA has been shown to more efficiently trigger innate immune and proinflammatory signaling (45). We estimated the amount of oxidized cell-free mt-DNA in dengue patients during the course of infection using the DNA damage competitive ELISA to detect the oxidized guanine species, 8-hydroxy-2′-deoxyguanosine, from DNA. We observed that the levels of cell-free oxidized DNA were high in dengue patients compared to the healthy controls and higher in the acute phase than in the convalescent phase (Fig. S12D). Overall, these findings imply that the release of mt-DNA into the bloodstream is a direct consequence of dengue infection, which wanes in patients during recovery or convalescence with the restoration of mitochondrial health.

## DISCUSSION

Acute liver and kidney manifestations are regarded as warning signs for progression to severe dengue as per the WHO-based criteria (46). *In vitro* studies show that DENV can infect different cell types, including epithelial cells, endothelial cells, muscle cells, dendritic cells, monocytes, macrophages, B cells, and mast cells (47, 48). Retrospective analysis of dengue in postmortem tissue samples from fatal dengue cases suggests the presence of DENV antigens such as viral envelope and NS3 proteins in many different tissues; however, infectious virus particles were obtained from the liver and PBMCs, suggesting that liver and immune cells may be the main regions of virus replication (48–51). The majority of DHF patients exhibit hepatocellular injury and manifest hepatomegaly, jaundice, and elevated liver enzymes resembling acute liver dysfunction. This suggests a direct effect of the DENV on hepatocyte viability and immune-mediated inflammation (hepatitis) and cytokine production in severe dengue (52, 53).

Being obligate parasites, viruses completely rely on the host cell machinery. Mitochondria play a central role in cellular metabolism and innate immune signaling and hence are primarily targeted by many viruses to create a conducive environment for their proliferation. The effect of the viruses on host cell mitochondria is either inflicted directly by viral proteins or through the physiological changes associated with viral infections such as oxidative stress, endoplasmic reticulum stress, hypoxia, and perturbed Ca^2+^ homeostasis (5). In agreement with previous observations (8, 9), we observed profound levels of mitochondrial injury evident by highly swollen and damaged mitochondria with an open permeability transition pore in infected hepatoma cells at later time points postinfection (Fig. 1 and Fig. S1). Ultrastructural analysis of infected hepatocytes in the liver tissue of fatal DHF cases suggests extensive mitochondrial injury evident by damaged and swollen mitochondria with distorted or damaged cisternae (50) and regions of hepatic necrosis and steatosis in liver tissue (50, 51).

Mitochondria are dynamic organelles and undergo constant cycles of fission and fusion and turnover through mitophagy and mitochondrial biogenesis to maintain mitochondrial quality control (26). When subjected to physiological stress or insults, mitochondria exhibit a graded response involving a change in morphology and dynamics through molecular players that regulate mitochondrial fission and fusion (54). Many viruses hijack the mitochondrial functions and mitochondrial dynamics to circumvent the host’s innate immune responses (55). DENV perturbed both the fission and fusion machinery by downregulating the expression of DRP1, a cytosolic GTPase that promotes mitochondrial scission and downregulation of the mitochondrial outer membrane fusion proteins mitofusin 1 and 2 (Fig. S3B), which was in agreement with previous reports (7–9). Mitochondrial fission enables both biogenesis of new mitochondria and the clearance of dysfunctional mitochondria via mitophagy. Distinct fission signatures predict mitochondrial degradation or biogenesis. Mitochondrial division at the periphery enables the segregation of damaged material to be subsequently eliminated by mitophagy, whereas division in the midzone primarily leads to the proliferation of mitochondria. Interestingly, both types of fission are mediated by DRP1, but midzone fission also involves constriction by the endoplasmic reticulum (ER) and actin (56). Due to the downregulation of DRP1 (Fig. S3A), the DENV-infected hepatic cells may lack the capacity to segregate damaged mitochondria from the rest of the healthy mitochondrial reticulum. Hepatitis C virus (HCV), a flavivirus like DENV, also induces profound levels of mitochondrial injury; however, upregulation of DRP1-mediated mitochondrial fission followed by mitophagy promotes viability of the infected cells and viral persistence (57). In contrast, HCV-infected cells silenced for Drp1 expression display swollen mitochondria and are defective for mitophagy and undergo cell death (57) similar to that observed in DENV-infected liver cells (Fig. 6). Mitophagy can occur through the PINK1-Parkin-dependent and -independent pathways and helps in the rapid elimination of damaged mitochondria by their sequestration and engulfment for subsequent lysosomal degradation (10, 11). The PINK1-Parkin axis flags the damaged mitochondria for elimination in a ubiquitin-dependent process. In unhealthy mitochondria, PINK1 import into the IMM is restricted, resulting in its accumulation on OMM, which then leads to subsequent activation and recruitment of Parkin. Active Parkin then adds polyubiquitin to the target proteins on OMM, which promotes the binding of autophagy adaptors such as optineurin, NDP52, and p62 and recruitment of autophagy machinery to the damaged mitochondria (28–30). Recently, a study on zika virus has shown that the virus protein NS5 antagonizes the PINK1-Parkin-dependent mitophagy by binding to the host protein Ajuba, which is required for PINK1 activation (58). We observed that DENV downregulates the expression of PINK1 and Parkin at both the transcript and protein levels, which may lead to a primary defect in flagging the damaged mitochondria for elimination by mitophagy (Fig. 4A to E and G). PINK1 has also been shown to promote mitochondrial fission by indirectly triggering DRP1 activity and its mitochondrial recruitment (59). Recent findings implicate several other ubiquitin E3 ligases in mitophagy regulation via the generation of polyubiquitin chains on their targets on the mitochondrial surface (11). However, the mitophagy reporter-based mitophagy flux assay in DENV-infected hepatic cells (Fig. 2) strongly indicates that mitophagy is not triggered and is also not induced upon treatment of the infected cells with the mitochondrial decoupler CCCP, suggesting that none of the mitophagy pathways are operative in DENV-infected hepatic cells.

Replenishment of mitochondrial mass in tandem with mitophagy is required to maintain the functional mitochondrial capacity of the cell. PPARγ and its coactivator PGC1α play a crucial role in mitochondrial biogenesis and turnover (34). PGC1α works in tandem with NRF2 and coactivates NRF1, and subsequently, the NRFs activate the transcription of nuclear-encoded mitochondrial proteins, including TFAM, which plays a central role in mitochondrial gene transcription and maintenance of genome copy numbers (32, 60). We observed that dengue downregulated the expression of PPARγ and its coactivator PGC1α and also downregulated the expression of NRF2 and TFAM (Fig. 5C). Determination of the rate of replenishment of mitochondrial mass using the mito-timer reporter (Fig. 5A and B) further validated that mitochondrial biogenesis is attenuated during dengue infection. In addition to their role in mitophagy, PINK1 and Parkin also govern mitochondrial biogenesis by regulating PGC-1α activation through degradation of Parkin interacting substrate (PARIS), a Krüppel associated box (KRAB) and zinc finger protein and transcriptional repressor of PGC-1α. PINK1 phosphorylates PARIS at S322 and S613, priming it for ubiquitination by Parkin and proteasomal degradation (61, 62). PINK1 and Parkin have also been shown to facilitate localized translation of nuclear-encoded mitochondrial protein to enable the replacement of damaged components (63). Due to the integral role of DRP1, PINK1, and Parkin in governing mitochondrial fission, mitophagy, and mitobiogenesis in a coordinated fashion, the downregulation of these crucial players during DENV infection (Fig. S3A and Fig. 4) may compromise mitochondrial quality, cellular injury, and necrosis.

Accumulation of damaged mitochondria may trigger innate immune signaling through the overproduction of ROS or the release of mt-DNA (15). Due to the shared common ancestry with bacterial DNA, mt-DNA is a potent danger signal that is recognized by the innate immune system and upregulates the inflammatory response (40, 44). Recent studies have shown that infection with RNA/DNA viruses that downregulates mitochondrial biogenesis causes the release of mt-DNA into cytosol and activation of the cellular antiviral response (31). Previous reports have shown the release of mt-DNA into the cytosol resulting in the activation of the toll-like receptor 9 (TLR9) and cyclic GMP-AMP synthase-stimulator of interferon genes (cGAS-STING) pathway, leading to the production of interferons in DENV-infected human dendritic cells (DCs) and lung epithelial cells (64–67). In addition, the activation of inflammasome and production of proinflammatory cytokines have been shown in various cell types upon dengue infection; however, the molecular cues that drive this remain obscure (68).

Freely circulating mt-DNA has been detected in the plasma and serum in many human diseases. Cellular stress and uncontrolled cell death are the keys to the release of mt-DNA in conditions with acute tissue injury, such as trauma, acute myocardial infarction, and sepsis (44). In acetaminophen overdose-associated liver injury, mt-DNA in the serum was found to be severalfold higher than normal; nonsurvivors had higher levels than survivors, and those with derangement in the liver enzymes had significantly higher mt-DNA levels than those who had normal liver enzymes, suggesting that the extent of mt-DNA release into the circulation depends on the extent of hepatocyte necrosis (69). Our observations suggest that a DENV-induced defect in mitochondrial quality control promotes necrosis in infected hepatic cells that can lead to the release of mt-DNA into the circulation and promote inflammation. We observed that the monocytes and human PBMCs challenged with culture supernatants of DENV-infected hepatic cells show activation of NLRP3 inflammasome and upregulation of proinflammatory cytokine expression (Fig. 9 and Fig. S12A). In addition, we also observed the release of mt-DNA at early time points postinfection, even before the onset of necrotic cell death, thereby implicating the role of exosome-dependent and -independent pathways in the release of mt-DNA (Fig. 7 and 8). Recent evidence suggests that exosome- or extracellular vesicle-mediated transfer of mitochondrial content alters the metabolic and inflammatory responses of recipient cells (70). Mitochondrial extracellular vesicles have been speculated to get released as microvesicles through plasma-membrane blebbing and/or through the formation of mitochondrion-derived vesicles (MDVs) that help in the delivery of mitochondrial components to other organelles or facilitate direct delivery of damaged mitochondrial components to the lysosomes (70). PINK1 and Parkin deficiency has also been directly implicated in mt-DNA release and inflammation. In alveolar epithelial cells, PINK1 deficiency-dependent mt-DNA release promoted activation of TLR9 and triggered profibrotic factor transforming growth factor β (TGF-β), which was rescued by PINK1 overexpression (71). Mice knocked out for PINK1 or Parkin display an increase in serum amounts of mt-DNA and the cytokines IL-6 and interferon-β (IFN-β) upon stress from mt-DNA mutation. However, blocking inflammation through inhibiting STING activity prevented neurodegeneration in Parkin knockout mice (72). PINK1-Parkin-pathway deficiency has also been implicated in autoimmune reactions and neurodegeneration through enhanced mitochondrial antigen presentation by regulating the pathway that relies on MDVs (73).

In the infected cells, dengue circumvents antiviral signaling at multiple levels: through evasion of viral RNA sensing, interference of retinoic acid-inducible gene (RIG-I) and mitochondrial antiviral-signaling (MAVS) protein and cGAS/STING pathways, and interference with interferon signaling (4, 74). Thus, more inflammatory damage may be inflicted by the release of DAMPs into the extracellular environment and circulation, leading to the trigger of inflammatory signaling in bystander naive cells. We observed the release of mt-DNA into the extracellular milieu from DENV-infected hepatic cells, and in agreement, we observed high levels of cell-free mt-DNA in the serum samples of dengue patients that declined in the convalescent stage of infection and strongly correlated with the degree of thrombocytopenia. However, we should contemplate with caution the source of mt-DNA in dengue patient serum samples, as platelets have also been shown to release mitochondria to promote inflammation in autoimmune disease and wound healing (75). Similarly, neutrophils have also been shown to release mt-DNA into the neutrophil extracellular traps (76). Alternatively, mt-DNA released in circulation during trauma has been shown to induce neutrophil extracellular traps (77).

However, further investigations are warranted to completely understand the mechanisms involved in cell-free mt-DNA in dengue patient serum samples and its implication for disease severity. As the innate immune pathways that thwart bacterial and viral infections cross-react with the mitochondrial components and invoke inflammation, the maintenance of mitochondrial health is pivotal to organismal health and well-being. Overall, this study enhances our understanding of mt-DNA-mediated inflammation during DENV infection due to the dysregulation of mechanisms governing mitochondrial homeostasis and quality control. It highlights the need to interrogate cell-type-specific interactions with the dengue virus to understand the mechanisms underlying disease pathogenesis and identify previously underrecognized factors in DENV immunopathogenesis. Our study also highlights the danger associated with chronic morbidities that create a proinflammatory state in paving the way for the onset of severe dengue disease and urges us to consider the impact of chronic liver pathologies on the progression to severe dengue.

## MATERIALS AND METHODS

### Virus propagation and titration.

All four dengue virus serotypes (Table S2) used in this study (DENV-1 West Pac 74, DENV-2 P23085 INDI-60, DENV-3 US/633798, and DENV-4 TVP-360) were propagated in C636 mosquito cells. The cells were infected at 0.01 MOI, and the culture medium containing the released virus was collected on day 5 postinfection. The viral titers in the culture supernatant were determined by the focus-forming unit (FFU) assay as described by Kim et al. (18). Briefly, the Vero cells were seeded at a density of 10,000 cells per well in a 96-well culture plate and 16 h later were infected with 10-fold serial dilutions of the virus-containing culture medium for 2 h; then, the inoculum was aspirated, followed by a 1× wash in phosphate-buffered saline (PBS), and replenished with fresh culture medium. Next, 72 h postinfection the cells were washed with 1× PBS and fixed in 4% paraformaldehyde, followed by blocking in 3% bovine serum albumin (BSA)-PBS for 1 h and overnight incubation at 4°C with dengue serotype-specific envelope monoclonal antibody. The next day the primary antibody was aspirated, followed by a 3× wash with PBS and incubation with anti-mouse Alexa Fluor 568-conjugated secondary antibody for 1 h at room temperature. The infection foci were visualized and counted using the Olympus DX58 fluorescence microscope, and infectious titers were calculated as FFU/mL culture supernatant.

### Cell culture and infection.

The Vero cells, THP1 monocytes, A549 cells, HEK cells, and C6/36 mosquito cells were obtained from the American Type Culture Collection (ATCC). Huh7 cells were a kind gift from Aleem Siddiqui, University of California at San Diego (UCSD). The Huh7 and Vero cells, A549 cells, and HEK cells were cultured in Dulbecco’s modified Eagle’s medium (DMEM) (Gibco) supplemented with 10% FBS, penicillin/streptomycin (10,000 units/mL), and 1× nonessential amino acids (NEAA) (Gibco). THP-1 and THP-1 ASC-GFP cells (InvivoGen) were cultured in RPMI 1640 (Gibco) medium supplemented with 10% FBS, 5 mM l-glutamine, 5% glucose, 1 mM sodium pyruvate, and penicillin/streptomycin (10,000 units/mL). All of these cell lines were kept at 37°C with 5% CO_2_. C6/36 cells were cultured in Leibovitz-15 medium supplemented with 10% FBS, 1× tryptone phosphate broth, and 1× antimycotic/antibiotic solution at 28°C. All the cells were regularly monitored for mycoplasma contamination.

For all of the experiments in this article, Huh7 cells were infected for 2 h with the various serotypes of dengue virus at 1 MOI in 2% FBS medium followed by 1× wash in PBS and replenishment with complete medium. The infected cells were then incubated for the respective times postinfection and processed as per the experimental requirements.

### Protease protection assay.

The protease protection assay was done as described previously by Nguyen et al. (78). Briefly, mock-infected, DENV-infected, or CCCP-treated Huh7 cells were homogenized in isotonic buffer containing 20 mM HEPES, pH 7.6, 220 mM mannitol, 70 mM sucrose, and 1 mM EDTA. The postnuclear supernatant was collected after 5 min of centrifugation at 500 × *g* at 4°C, and protein quantification was performed using the bicinchoninic acid (BCA) technique. Equal amount of protein samples were subjected to no treatment or treated with 25 g/mL proteinase K on ice for 10 min or with proteinase K plus 0.2% Triton X-100 (vol/vol). The proteinase K activity was subsequently inhibited by the addition of 1 mM phenylmethylsulfonyl fluoride (PMSF). Posttreatment, the samples were subjected to Western blot analysis to determine the protection status of their respective mitochondrial proteins.

### Immunofluorescence assay.

Mock- or dengue-infected cells grown on sterile glass coverslips in a 6-well plate after the required incubation period were washed in PBS and fixed in 4% paraformaldehyde. Later the cells were blocked and permeabilized for 1 h in PBS with 0.1% Triton X-100, 1% BSA, and 5% FBS. After blocking, the cells were incubated overnight at 4°C with primary antibody solution. Subsequently, the cells are washed 3 times in PBS, followed by incubation with the Alexa-Fluor-conjugated secondary antibodies (Invitrogen). Finally, the coverslips were mounted on Prolong gold antifade 4′,6-diamidino-2-phenylindole (DAPI; Thermo Fisher Scientific). The images were captured using a 60× lens objective on a Leica TCS SP8 confocal microscope. ImageJ software was used for image analysis and quantitation.

### Reporter assays.

To study the effect of dengue on mitophagy, autophagy, and mitochondrial biogenesis, we used pAT016 (p-mito-mRFP-EGFP) mitophagy reporter, ptfLC3 (p-LC3-EGFP-C1-mRFP) autophagy reporter, and pMito-timer, respectively. Huh7 cells were grown on sterile glass coverslips at 60% confluence. The next day, cells were transfected with the reporter plasmids, and 16 h posttransfection, cells were either mock or Dengue infected at an MOI of 3 for the indicated times. To check the induction of mitophagy (in the mitophagy reporter assay), transfected-infected cells were treated with 20 μM CCCP for 12 h. After requisite the times, cells were fixed in 4% paraformaldehyde and probed with Dengue-specific antibody for further analysis through confocal microscopy.

### Promoter reporter assay.

The Huh7 cells were cotransfected with the PGC1α promoter-reporter and renilla luciferase vector (used for normalizing transfection efficiency). Then, 16 h posttransfection the cells were mock infected or infected with the dengue serotype at an MOI of 3. Next, 48 h postinfection the firefly and renilla luciferase activity was determined using the dual-luciferase reporter system (Promega) as per the manufacturer’s instructions. Briefly, the cells were washed with PBS and lysed for 15 min at room temperature in 1× passive lysis buffer. Cell lysates were then centrifuged at 4°C for 5 min at 12,000 rpm; 30 μL of cell lysate was mixed with 100 μL LAR II reagent, and firefly activity was detected in a VICTOR Nivo multimode reader, followed by the addition of Stop & Glo reagent and detection of renilla luciferase activity.

### Mitochondrial isolation.

The mitochondrial fractions were isolated from whole-cell lysates as previously reported (79). Briefly, the cells are homogenized in an isotonic buffer comprising 225 mM mannitol, 75 mM sucrose, 0.1 mM EGTA [ethylene glycol-bis(β-aminoethyl ether)-*N*,*N*,*N′*,*N′*-tetraacetic acid], and 30 mM Tris-HCl, pH 7.4. After that, homogenates were centrifuged at 1,000 × *g* for 5 min to remove cell debris and the nuclear pellet. The postnuclear supernatant was centrifuged at 7,000 × *g* for 10 min to obtain a crude mitochondrial pellet. The resulting mitochondrial pellet was washed once and resuspended in the mitochondrial resuspension buffer (MRB), which contained 250 mM mannitol, 5 mM HEPES (pH 7.4), and 0.5 mM EGTA. The postmitochondrial supernatant was subjected to ultracentrifugation at 100,000 × *g* for 1 h to pellet the microsomal fraction and obtain a pure cytosolic fraction. An equal amount of protein lysate from each fraction was subjected to Western blot analysis or used for mt-DNA isolation.

### mt-DNA isolation.

mt-DNA was isolated from cell culture supernatant, exosomes, or patient plasma using the Thermo Fisher Scientific MagMAX cell-free DNA isolation kit according to the manufacturer’s instructions. In brief, an adequate amount of MagMAX cell-free DNA lysis/binding solution and magnetic beads were added to 200 μL of patient plasma and shaken for 10 min at medium speed on the vortex mixer, followed by pelleting of beads against a DynaMag magnet for 5 min. Without disturbing the beads, the supernatant was removed and washed with 500 μL of MagMAX cell-free DNA wash solution, followed by an 80% ethanol wash. Following the ethanol wash, the beads were air-dried for 2 to 3 min to evaporate ethanol remnants, and 50 μL of MagMAX cell-free DNA elution solution was added to the magnetic beads to extract the mt-DNA, which was then subjected to qPCR using mitochondrial genome-specific primers (Table S1). mt-DNA from the cytosol and enriched mitochondria suspension obtained from cultured cells were performed using the DNeasy blood and tissue kit (69504, Qiagen) according to the manufacturer’s instructions.

### Viral RNA extraction and estimation.

The supernatant of dengue (serotypes 1 to 4)-infected cells was used for viral RNA isolation using the TRIzol reagent (Life Technologies, USA) according to the manufacturer’s procedure. Viral genome copies were quantified using qRT-PCR in the Quant Studio 6 real-time PCR machine using the TaKaRa PrimeScript one-step real-time PCR (RT-PCR) kit (RR055A) according to the manufacturer’s instructions. The DENV primers/probes specified in Table S1 were used.

### Plasmids.

The dual fluorescent reporter plasmids pAT016 (p-mito-mRFP-EGFP, a kind gift from Andreas Till, and pTF-LC3, a kind gift from Tamotsu Yoshimori) were used to monitor mitophagy and autophagy flux, respectively (18, 19). pMito-timer (Addgene no. 52659) was used to track mitochondrial biogenesis. PGC-1 alpha promoter 2-kb luciferase plasmid (Addgene no. 8887) and renilla luciferase plasmid (Promega no. E2231) were obtained from Addgene and Promega, respectively. The dengue 2 nonstructural protein constructs in the pLVX-IRES-ZsGreen1 backbone were a gift from Ali Amara and have been described previously (80). The plasmids were used to subclone the nonstructural proteins into the pCMV3Tag3A vector by restricted digestion with either SpeI-BamHI (NS1, NS2B, and NS3) or EcoRI-BamHI (NS2A, NS5, NS4A, or NS4B) and ligation into pCMV3Tag3A. The dengue 2 structural proteins were cloned into the pCMV3Tag3A vector using gene-specific primers with suitable restriction sites. The cloning primers for PCR synthesis of envelope, prM, and capsid regions used are shown in Table S1. The PCR fragments were restriction digested, gel purified, and ligated into the pCMV3Tag3A vector. The positive clones were confirmed by DNA sequencing.

### Mitochondrial membrane potential and ROS detection.

Mitochondrial membrane potential-specific JC-1 dye (1 μg/mL; 15 min) and mitochondrial superoxide-specific MitoSOX dye (Invitrogen no. M36008) (100 nM, 20 min) were used to evaluate mitochondrial membrane potential and superoxide production as per the manufacturer’s instructions. JC-1 dye is a lipophilic cationic dye which naturally exhibits green fluorescence as a monomer; however, when it enters mitochondria JC-1 accumulates, resulting in the formation of reversible complexes called J aggregates, which exhibit red fluorescence instead of green. In brief, the cells were stained with fresh medium containing the respective dyes after the incubation period postinfection with dengue and were incubated further for 15 to 20 min. After a 2× wash in PBS, the cells were trypsinized and resuspended in PBS and analyzed using a FACSCalibur device (Beckton and Dickinson). The FlowJo program was used to evaluate the data. For live imaging of JC-1 dye-stained cells, the cells grown on optically clear-bottom dishes were subjected to treatments as described above, and live imaging was performed after the requisite incubation period with an Olympus IX83 inverted fluorescence microscope with a 40× lens objective.

### Monocyte/macrophage stimulation assay.

Huh7 cells were mock- or dengue serotype 1 virus-infected with 1 MOI for 48 h and 60 h. The supernatant was collected and preclarified by centrifugation to remove cell debris. The clarified culture supernatants were used to challenge THP1 monocytes, THP-ASC-GFP monocyte-derived macrophages, and purified human PBMCs for 8 h. Direct infection with dengue 1 viruses for 2 h or treatment with LPS (1 μg/mL) for 3 h followed by nigericin (10 μM) for 15 min was done to serve as a direct infection and inflammation-positive control. DNase I (160 U/mL) was used to digest mt-DNA by incubating DENV-infected supernatant at 37°C for 30 min. After the treatments, the control cells were also incubated up to 8 h prior to collection of the cells and culture supernatants for further analysis. For ASC-GFP speck analysis, the images were acquired using 20× lens objective in an Olympus IX83 fluorescence microscope in all of the above-mentioned circumstances to identify inflammasome-activated cells. The clarified culture supernatants were used for IL-1β and IL-18 ELISA. The cells were collected and subjected to RNA isolation by the TRIzol method. Purified RNA was used for gene expression analyses.

### Exosome isolation.

Exosomes were isolated using the total exosome isolation kit (Invitrogen, catalog [cat.] no. 4478359) according to the manufacturer’s instructions. To summarize, the culture supernatants from mock- and DENV-infected Huh7 cells were clarified by centrifugation at 5,000 rpm for 5 min to remove any cell debris. Then, 500 μL of total exosome isolation reagent was added to 1 mL of clarified culture supernatant and thoroughly mixed by vortexing before incubation at 4°C overnight. Following incubation, samples were centrifuged at 10,000 × *g* for 1 h at 4°C, and the supernatant was discarded. Pelleted exosomes were dissolved in 100 μL PBS and utilized to isolate mt-DNA.

### Cell death analysis by fluorescence-activated cell sorter (FACs).

Cell death was determined using annexin V/propidium iodide (PI) staining as directed by the manufacturer (Invitrogen no. V13241). Huh7 cells were infected with the serotypes of dengue at 1 MOI, and 60 h postinfection the cells were trypsinized and collected in 1× cold PBS. After cell density was determined, about 1 × 10^6^ cells were centrifuged and resuspended in 100 μL of 1× annexin V binding buffer. The cell suspension was then incubated for 15 min at room temperature with 5 μL Alexa Fluor 488 annexin V (component A) and 1 μL 100 μg/mL PI working solution. Following incubation, 400 μL of 1× annexin V binding buffer was gently mixed into the cell suspension, and the mixture was immediately placed on ice. The stained cells were analyzed using a FACSCalibur device (Beckton and Dickinson), and the data were evaluated using FlowJo software.

### Sample collection.

Peripheral blood samples (5 mL) from laboratory-confirmed dengue patients were collected on the day of enrollment and in the convalescent phase prior to discharge from the hospital. Informed consent was taken in every case. Plasma was prepared by centrifugation at 3,000 rpm for 10 min and stored as 500-μL aliquots in −80°C deep freezers until further use. The samples were reconfirmed for dengue infection by NS1/IgM rapid antigen test and PCR. Only the confirmed positive samples were considered further for the study.

### Ethics statement.

The protocol was approved by the Ethical Committee of the Institute of Life Sciences, India (48/HEC/16).

### Image analysis.

The ImageJ macro Mitochondrial Analyzer was used for morphological analysis on mitochondria. The study was performed per-mito parameters, with the “form factor” determining the mitochondrial length and “counts” indicating the number of mitochondria per cell. The Mitochondria Morphology plugin was used to account for mitochondrial swelling. The area/perimeter ratio was standardized to circularity in order to measure swollenness. The colocalization threshold option of the Colocalization plugin was used to calculate colocalization spots. Ratiometric analysis was performed for two distinct channels by first thresholding the pictures for both the channels after eliminating the background and then using the analyze option to obtain area integrated intensity and mean gray value. Total cell fluorescence was calculated by the following formula corrected total cell fluorescence (CTCF) = integrated density − (area of selected cell × mean fluorescence of background reading) (81).

### Statistical analysis.

All experiments were carried out as two or three independent replicates, and the data presented are the mean ± the standard error of the mean. Throughout the article, statistical analysis was done using the Student’s *t* test, one-way analysis of variance (ANOVA), or two-way ANOVA in GraphPad Prism 9, as per the experimental requirement based on single- or multiple-parameter analysis to assess the significance level. A *P* value of <0.05 was regarded as significant.
