# Supplementary material for: Defective Mitochondrial Quality Control during Dengue Infection Contributes to Disease Pathogenesis
Source: J Virol. 2022 Oct 5;96(20):e00828-22. doi: 10.1128/jvi.00828-22 (PMC9599662; doi:10.1128/jvi.00828-22)
Supplement: Supplemental file 1 — Supplemental methods, Tables S1 and S2, and Fig. S1 to S12. Download jvi.00828-22-s0001.pdf, PDF file, 12.2 MB [file jvi.00828-22-s0001.pdf]

Supplementary Information

Supplementary Methods

Total RNA extraction and gene expression analysis: The total cellular RNA extraction from mock- and infected cells was performed using the Trizol (Invitrogen, USA) method as per the manufacturer's protocol. The Nanodrop Spectrophotometer was used to evaluate the quality and amount of RNA. Takara Primescript^{TM} 1st strand cDNA synthesis kit (6110A) with Oligo dT primers was used to synthesise cDNA from 2 ug of total RNA. Gene expression analysis was performed with PowerUp^{TM} SYBR^{TM} Green Master Mix (A25741) in Applied Biosystems Quant Studio 6 using the gene-specific primers provided in **S1 Table**.

Western blot Analysis: Mock or dengue-infected cells were collected and lysed in RIPA buffer supplemented with a cocktail of protease and phosphatase inhibitors. Lysates were briefly sonicated and centrifuged at 13,000 rpm for 20 minutes at 4°C to obtain the clear whole cell lysates. Protein concentration was estimated using the Pierce BCA Protein Assay kit. An equal amount of protein obtained from various samples was subjected to SDS-PAGE and Western blot. The blot membranes were blocked with 3% BSA blocking buffer, followed by overnight incubation with respective primary antibody and corresponding HRP-conjugated secondary antibody (1:5000 dilution, Promega) for one hour at room temperature. Blots were developed using the Clarity Western ECL substrate (Bio-Rad) and images were acquired with the Chemi-Doc MP Imaging System (Bio-Rad). All the antibodies used in this study are enlisted in **S2 Table.**

Caspase Assay: Caspase-Glo® 1 or Caspase-Glo® 3/7 assay kits (Promega, G9951 and G8090) was used according to the manufacturer's instructions to measure Caspase-1 activity. In a 96 well plate, cell lysates with similar protein concentrations were added to each well, followed by the addition of 100uL of either Caspase-Glo 1 or Caspase-Glo 3/7 reagent and incubated at room temperature for 2h prior to luminescence detection in the VICTOR Nivo multimode reader.

Enzyme-linked immunosorbent assay (ELISA): ELISA for Human IL-1 beta (Invitrogen # 88-7261), Il-18 (Invitrogen #BMS267-2) and DNA Damage (Invitrogen # EIADNAD) was done as per manufacturer’s instruction. Readings were acquired in VICTOR Nivo multimode reader at 450 nm.

PBMC’s isolation: 5 mL of peripheral blood was collected and carefully poured into the CPT cell preparation tube (BD Bioscience, # 362753) for isolation of PBMCs. The tubes were

centrifuged at 1600g for 15mins and the top 3/4th layer of plasma was discarded, while the remaining 1/4th was gently mixed with the PBMC layer and transferred to a separate 15ml tube. This tube was centrifuged for 5mins at 300g to pellet the PBMCs which were subsequently washed once in RPMI media and pelleted. The cell pellet was resuspended in 1ml RPMI, and cell density was determined. Around 2 x10^6 cells were taken per condition for the dengue-infected Huh7 culture supernatant challenge experiment.

Table S1: Primers used in this study

**Gene specific primers**	
MFN1 R	TTGGAGAGCCGCTCATTCACCT
MFN2 F	GTGGAATACGCCAGTGAGAAGC
MFN2 R	CAACTTGCTGGCACAGATGAGC
PARK2 F	CCAGAGGAAAGTCACCTGCGAA
PARK2 R	GTTCGAGCAGTGAGTCGCAATC
PINK1 F	GTGGACCATCTGGTTCAACAGG
PINK1 R	GCAGCCAAAATCTGCGATCACC
DRP1 F	GCGAACCTTAGAATCTGTGGACC
DRP1 R	CAGGCACAAATAAAGCAGGACGG
Mff F	CCAAACGCTGACCTGGAAC
Mff R	TTTCCTGCTACAACAATCCTCTCC
TOMM20 F	GAAGAGCAGAGCCTCAAAAGGC
TOMM20 R	GTTCTCCCATCCGTACCTCTTG
GAPDH FP	GTCTCCTCTGACTTCAACAGCG
GAPDH RP	ACCACCCTGTTGCTGTAGCCAA
ASC FP	CGCGAGGGTCACAAACGT
ASC RP	TGCTCATCCGTCAGGACCTT
NLRP3 FP	GGACTGAAGCACCTGTTGTGCA
NLRP3 RP	TCCTGAGTCTCCCAAGGCATTC
IL1B F	CCACAGACCTTCCAGGAGAATG
IL1B R	GTGCAGTTCAGTGATCGTACAGG
IL18R1 F	GGAGGCACAGACACCAAAAGCT
IL18R1 R	AGGCACACTACTGCCACCAAGA
CASPASE1 FP	GGGTCGCTTTTGGGATTACCTG
CASPASE1 RP	CAACTCCTTCATGGTCTCGTCC
PHB1 F	GCGTGGTGAACTCTGCCTTA
PHB1 R	TGTACCCACGGGATGAGAAA
BNIP3 F	GCTCCCAGACACCACAAGAT
BNIP3 R	TGAGAGTAGCTGTGCGCTTC
Rubicon FP	CAGATTCTGCTGCCTCTTCC
Rubicon RP	AGTGTCTGCCCCTCTGAGAA
UVRAG FP	ACAAACTGACGGAAAAGGAGAG

UVRAG RP	CCATGTTGATATCTTAGCTGTGC
GABARAB FP	GTAGCAACACGGTTCGTGAATA
GABARAB RP	AATCAGACGGAGGTGACTTGTT
LC3 A FP	TCCTGGACAAGACCAAGTTTTT
LC3 A RP	GTGAAAGGCTGGGAATCATTCT
LC3 B FP	TGGCCCTTAGTAATGCTTCTGT
LC3 B RP	TAGGTTGTGAAACTGACACCCA
LC3 C FP	GTAAGACACCACTGGACTTCCG
LC3 C RP	CCAAAATAAAACTGCCAAACGA
NDP52 FP	ACGCAAGGACTGGATTGGCATC
NDP52 RP	GGATTTCCTGCTGTGTGGCTGA
OPA FP	GCTGCTCCTCTAACAATGGCAC
OPA RP	TCTTCCAGGAGCAAACACTGCC
NIX FP	CCTCGTCTTCCATCCACAAT
NIX RP	GTCCCTGCTGGTATGCATCT
OPTINEURIN FP	AGGTGGAGAGACTTGAAGTCGC
OPTINEURIN RP	TCCTCGCTGTCTGCTTCTCAGT
p62 FP	TGTGTAGCGTCTGCGAGGGAAA
p62 RP	AGTGTCCGTGTTTCACCTTCCG
**mt DNA specific PCR primers**	
mt 16s rRNA FP	GCCTTCCCCCGTAAATGATA
mt 16s rRNA RP	TTATGCGATTACCGGGCTCT
ND2 FP	AGCAGGCAGTTGAGGTGGAT
ND2 RP	TTGGGCAAAAAGCCGGTTAG
ATP6 FP	GCCACAACTAACCTCCTCGG
ATP6 RP	TAGGGTGGCGCTTCCAATTA
ND5 FP	TTCATCCCTGTAGCATTGTTCG
ND5 RP	GTTGGAATAGGTTGTTAGCGGTA
**DENV specific primers and probe**	
DENV 1 FP	CATCAGCTATGGGCTACCTTG
DENV 1 RP	GAAGTCGTGGACAGGCATAAA
DENV 1 Probe	FAM-TGCACTATGCATGGAAGACAATGGC-BHQ
**Cloning primers**	
BamHI **Capsid** FP	CGGGATCCATGAATAACCAACGGAAAAAGGCGAGAAACAC
EcoRI **Capsid** RP	GGAATTCTGCCATCACTGTAGGAATCAGCATGAT
SacII **PrM** FP	TCCCCGCGGATGTTTCATTTGACCACACGCAACGGAGAACCAC
HindIII **PrM** RP	CCCAAGCTTTGTCATTGAGGGAGCGATGGCTGTCAG
EcoRI **Env** FP	GGAATTCATGCGCTGCATAGGAATATCAAATAGGGAC
XhoI **Env** RP	CCGCTCGAGGGCCTGCACCATAACTCCCAAGTATAGTG

Table S2: List of reagents used in this study

Reagents	Source	Catalog #
**Cell Lines**		
Huh-7	Kind gift from Dr. Aleem Siddiqui, UCSD	N/A
Vero	ATCC	CCL-81
THP-1	ATCC	TIB-202
THP-ASC-GFP	Invivogen	THP-ASC-GFP
**Virus strain**		
Dengue-1 (WP 74)	Kind gift from Dr. De Silva, University of North Carolina, USA	U88535
Dengue-2 (P23085 INDI-60)	Kind gift from Dr. Manjula Kalia, RCB, Faridabad	KJ918750
DENV-3/USA/633798/1963	NIV Pune	AFZ40124
Dengue-4 (TVP-360)	Kind gift from Dr. De Silva, University of North Carolina, USA	KU513442
**Antibodies**		
Goat anti-DENV-1	Santa Cruz	SC325013
Goat anti-DENV-2	Santa Cruz	SC325014
Goat anti-DENV-3	Santa Cruz	SC325015
Goat anti-DENV-4	Santa Cruz	SC325021
Mouse anti-COX-2	Santa Cruz	SC514489
Mouse anti-GAPDH	Santa Cruz	SC25778
Mouse anti-Nrf2	Santa Cruz	SC365949
Mouse anti-Optineurin	Santa Cruz	SC166576
Mouse anti-Tim23	Santa Cruz	SC 514463
Mouse anti-TOM20	Santa Cruz	SC 17764
Mouse anti-ASC	Santa Cruz	SC514414
Mouse anti-mtTFA(C-9)	Santa Cruz	SC-376672
Mouse anti-PARP1	Santa Cruz	SC 8007
Rabbit anti-Caspase-3	Cell Signaling Technologies	9662S
Rabbit anti-DRP 1	Cell Signaling Technologies	8570S
Rabbit anti-PDRP 1	Cell Signaling Technologies	4494S
Rabbit anti-HA	Cell Signaling Technologies	3724 S
Rabbit anti-IL-1β	Cell Signaling Technologies	12703S
Rabbit anti-LAMP 1	Cell Signaling Technologies	9091S
Rabbit anti-MFN1	Cell Signaling Technologies	14395
Rabbit anti-MFN2	Cell Signaling Technologies	11925S
Rabbit anti-MFF	Cell Signaling Technologies	84580S
Rabbit anti-PMFF	Cell Signaling Technologies	49281S
Rabbit anti-NDP52	Cell Signaling Technologies	9036
Rabbit anti-NLRP3	Cell Signaling Technologies	15101S
Rabbit anti-OPA-1	Cell Signaling Technologies	67589S
Rabbit anti-PGC1α	Cell Signaling Technologies	2178S
Rabbit anti-PINK 1	Cell Signaling Technologies	6946S

Rabbit anti-TOM 20	Cell Signaling Technologies	42406S
Rabbit anti-PINK1	Cell Signaling Technologies	6946
Rabbit anti-VDAC	Cell Signaling Technologies	4661S
Rabbit anti-β-actin	Cell Signaling Technologies	4970
Rabbit anti-CASPASE- 1	Cell Signaling Technologies	2225S
Rabbit anti-COX-IV	Cell Signaling Technologies	4844S
Rabbit anti-PPARγ	Cell Signaling Technologies	2443
Rabbit anti-LC3B	Sigma-aldrich	L7543
Rabbit anti-Hsp60	Bio-Bharati	BB-AB0209
Rabbit anti-Dengue Virus PrM	Gentex	GTX128092
Rabbit anti- Dengue Virus NS3	Gentex	GTX124252
Rabbit anti-Parkin	Abcam	ab15954
Mouse anti-PINK1	Abcam	ab75487
Mouse anti-ds DNA	Abcam	ab27156

Supplementary Figure Legends:

Figure S1: (A) Representative confocal images of Huh7 cells either mock-infected or infected with dengue serotype 1 for 24h, 48h, and 60h post-infection. Cells were immunostained for mitochondrial marker TOMM20 (green). The adjacent grayscale images represent the 2D trace of the tubular mitochondrial network obtained by the Skeletonize 2D/3D plugin of ImageJ. **(B)** Graph depicting the quantification of the % of cells displaying highly elongated mitochondria in mock- and dengue-infected cells at indicated time post-infection. **(C)** Bar graph depicting the mitochondrial superoxide levels in mock- and dengue-infected Huh7 cells 48h post-infection. H_2O_2 was used as a positive control to induce mitochondrial superoxide levels. Statistical analysis was done using one-way ANOVA ***P < 0.001, ****P < 0.0001.

Figure S2: Dengue infection induces mitochondrial damage in A549 and HEK cells

Confocal microscopy images of A549 **(A)** and HEK cells **(B)** infected with dengue-2 serotype at indicated time points. The cells were immunostained with dengue-2 serotype-specific anti-envelope, and mitochondria-specific anti-TOMM20 antibodies. Nuclei were counterstained with DAPI. Scale bar shown is 10μm. Around 30-40 cells per condition were quantified for the various features of the mitochondria such as number, length, and circularity. The quantification is depicted as % of mock for average mitochondrial number per cell, mitochondrial footprint, and circularity **(A-B).** Bar graph depicting the mitochondrial superoxide levels in mock- and dengue-2 infected A549 **(C)** and HEK cells **(D)** cells 48h post-infection. H_2O_2 was used as a positive control to induce mitochondrial superoxide levels. Data presented is the mean ± SEM of three independent experiments. Statistical analysis was done using one way ANOVA (B-D,G) or two-way ANOVA (F). *P < 0.05, **P < 0.01, ***P < 0.001, ****P < 0.0001.

Figure S3: Dengue perturbs mitochondrial fission and fusion machinery.

(A) Western blot analysis of the indicated mitochondrial fission proteins in Huh7 cells either mock-infected or infected with respective dengue serotypes for 24h, 48h and 60h post-infection. NS3 is used as an infection marker and actin as the internal loading control. The bar graphs depict densitometry analysis of bands from three Western blots for the respective proteins. **(B)** Western blot analysis of the indicated mitochondrial fusion proteins in Huh7 cells either mock-infected or infected with respective dengue serotypes for 24h, 48h and 60h post-infection. PrM is used as an infection marker and actin as the internal loading control. The bar graphs depict densitometry analysis of bands from three Western blots for the respective proteins. **(C)** Heatmap representing the transcript level of the indicated mitochondria fission and fusion genes in Huh7 cells infected with various dengue serotypes for indicated time post infection. Data are mean ± SEM from three independent experiments. Statistical analysis was done using one-way ANOVA. ns = non-significant, *$P < 0.05$, **$P < 0.01$.

Figure S4: Dengue infection inhibits mitophagy

(A) Huh7 cells transfected with the mitophagy reporter p-mito-mRFP-EGFP were treated with CCCP, 40h post-transfection and stained with the lysosome marker LAMP1. Representative confocal image of cells harbouring the mitophagy reporter. White arrows in the zoomed inset depict colocalization between lysosomes (LAMP1 positive) and only red mitochondria (mitochondria delivered to lysosomes). **(B)** Confocal images of Huh7 cells either mock-infected or infected with respective serotypes of dengue for 48h and stained with lysosome and mitochondrial markers LAMP1 (green) and TOMM20 (red). Dengue envelope (cyan) is used as a marker of infection. **(C)** Confocal images of Huh7 cells either mock-infected or infected with respective serotypes of dengue for 48h and further treated with CCCP to induce mitophagy. Fixed cells were stained with lysosome and mitochondrial markers LAMP1 (green) and TOMM20 (red). Dengue envelope (cyan) is used as a marker of infection. Pixel-density images in the right represent the colocalized spots between mitochondria-lysosomes obtained by the merge of red and green channels. **(D)** Confocal images of Huh7 cells either mock-infected or infected with dengue1 for 48h. Fixed cells were stained with LC3B (green) and mitochondrial markers TOMM20 (red). Zoomed inset show regions of colocalization between the green and red channels represented by yellow spots.

Figure S5: (A) Densitometry analysis of bands representing the proteins shown in figure 2F from three independent Western blots. **(B)** Western blot analysis of the indicated mitochondrial

proteins in mock- and dengue-infected Huh7 cells, 40h post-infection untreated or treated with 125nm of bafilomycin-A for 8h to inhibit lysosomal degradation. NS3 was used as an infection marker and actin as an internal loading control. Statistical significance was determined using one-way ANOVA. Only significant variations are indicated in the graph. *$P < 0.05$.

Figure S6: Protease protection assay

To check the status of mitophagosome formation in dengue infected Huh7 cells we performed the mitophagosome protease protection assay. **(A)** Schematic representation of the protease (proteinase K) protection assay. Mitochondria engulfed in the phagosomes (mitophagosome) are protected from proteinase K treatment and treatment with the detergent triton x100 (TX100) leads to proteinase K sensitivity due to membrane lysis **(B)**. Western blot analysis of the mitochondrial proteins TOMM20, OPA1, and HSP60 in the crude cytosol fractions obtained from mock, CCCP-treated, and dengue infected Huh7 cells subjected to the indicated treatments.

Figure: S7: Effect of individual dengue virus protein on mitophagy flux

(A) Huh7 cells were co-transfected with the mitophagy reporter p-mito-mRFP-EGFP and respective pCMV3Tag3A vectors expressing the individual dengue virus proteins to determine the effect of their ectopic expression on the status of mitophagy flux. Representative confocal images of cells co-expressing the respective dengue virus proteins (anti-HA antibody) and mitophagy reporter. **(B)** Quantification of the average number of red mitochondria per cell in cells harbouring the respective dengue protein and mitophagy reporter. **(C)** Western blot analysis depicting the expression level of each individual dengue viral protein detected by anti-HA and anti-FLAG antibodies in cell lysates obtained from Huh7 cells transfected with the respective expression vectors. Data are mean ± SEM of three independent experiments. Statistical analysis was done using one-way ANOVA. ns = non-significant, *$P < 0.05$, ****$P < 0.0001$.

Figure S8: (A) Densitometry analysis of cleaved PINK1 (p44 band), and total PINK1 (p44 + p64), Parkin, NDP52 and optineurin bands to indicate their relative expression status in respective conditions. **(B)** Western blot analysis of PINK1 and Parkin in the purified mitochondrial and cytosol fractions obtained from mock-infected or dengue serotype 1 or 2 infected Huh7 cells at 24h and 48h post-infection. CCCP treated Huh7 cells fractions were

used as a positive control for PINK1 and Parkin translocation to mitochondria. VDAC and GAPDH were used as markers to indicate the purity of the mitochondrial and cytosol fractions. Statistical significance was determined using one-way ANOVA. Only significant variations are indicated in the graph. *P < 0.05, **P < 0.01, ***P < 0.001

Figure S9: Western blot **(A)** and transcript **(B)** analysis of BNIP3, BNIP3L/Nix, and Prohibitin in Huh7 cells either mock-infected or infected with respective dengue serotypes for 24h, 48h and 60h post-infection. NS3 is used as an infection marker and actin as the internal loading control. Western blot analysis of PINK1 & Parkin **(C)** and NDP52 & optineurin **(D)** in Huh7 cells either mock-infected or infected with various dengue serotypes for 40h followed by no treatment or treatment with 125nm of bafilomycin-A for 8h to inhibit lysosomal degradation. PrM and NS4B were respectively used as an infection marker and actin as an internal loading control

Figure S10: (A) Densitometry analysis of bands representing the proteins shown in figure 5C from three independent Western blots. **(B)** Heat map representing the transcript levels of PGC1α and NRF2 in Huh7 cells infected with respective serotypes of dengue at indicated time points post-infection in comparison to mock-infected cells. **(C)** Densitometry analysis of bands representing cleaved PARP-1 (A), caspase 3 p17 band (B) and Caspase1 p20 band (C) from three independent Western blots as shown in Figures 6C & 6E. Statistical significance was determined using one-way ANOVA. ns= non-significant, *P < 0.05. Statistical significance was determined using one-way ANOVA. Only significant variations are indicated in the graph. *P < 0.05, **P < 0.01, ***P < 0.001

Figure S11: Dengue virus infection leads to mt-DNA release in A549 and HEK cells

mtDNA isolated from the cytosol fractions **(A)** and pre-clarified cell culture medium **(B)** of the DENV-2 infected A549 cells was subjected to qRT-PCR. Bar graph depicting the relative levels (fold change with respect to mock) of ATP6 and mt-16S RNA. mtDNA isolated from the cytosol fractions **(C)** and pre-clarified cell culture medium **(D)** of the DENV-2 infected HEK cells was subjected to qRT-PCR. Bar graph depicting the relative levels (fold change with respect to mock) of ATP6 and mt-16S RNA. Data are mean ± SEM of three independent experiments. Statistical significance was determined using one way ANOVA. ns= non-significant, *P < 0.05, **P < 0.01, ***P < 0.001, ****P < 0.0001.

Figure S12:

Human PBMCs were challenged for 8h with clarified culture supernatants obtained from dengue serotype 1 infected (D1) Huh7 cells at 48h and 60h post-infection. Challenge with culture-supernatant from mock-infected Huh7 cells, LPS+Nigericin, and direct infection with dengue virus 1 were used as negative, positive, and infection control. **(A)** The graph depicts relative fold change in the transcript level of IL-1β, IL-18 and caspase-1 in PBMCs challenged with indicated conditions with respect to untreated PBMCs. **(B)** The graph depicts relative fold change in the transcript level of IL-1β, IL-18 and caspase-1 in monocytes challenged with clarified culture supernatants obtained from dengue serotype 1 infected (D1) Huh7 cells at 48h or similar culture supernatants pre-treated with DNAse 1 (160U/ml, 37ºC for 30 mins). **(C)** Bar graph depicting the quantification of the number of ASC-GFP puncta/specks per cell, 8h post-challenge of ASC-GFP THP1 cells with indicated treatments. **(D)** mt-DNA isolated from the plasma of dengue patients (n=10) collected at the acute and convalescent phase and age-matched healthy controls (n=9) was subjected to DNA damage ELISA. The graph depicts the relative quantity of 8-OHdG levels (ng/mL) in the indicated group of samples. Data are mean ± SEM from three independent experiments. Statistical significance was determined using one-way ANOVA. ns= non-significant, *P < 0.05; **P < 0.01;***P < 0.001,****P < 0.0001.

S1 Fig

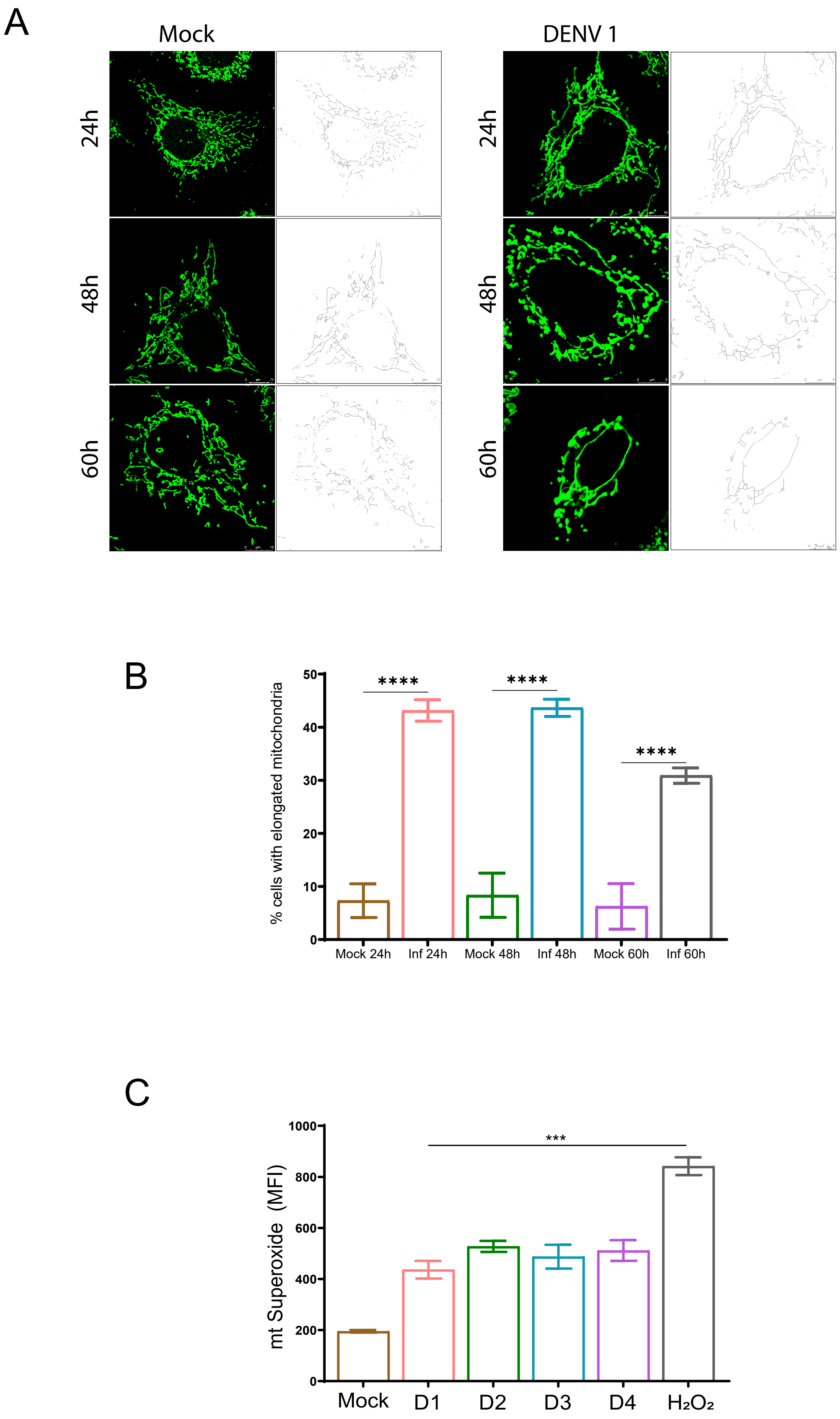
A
Mock
DENV 1
24h
48h
60h
B
% cells with elongated mitochondria
50
40
30
20
10
0
****
****
****
Mock 24h
Inf 24h
Mock 48h
Inf 48h
Mock 60h
Inf 60h
C
mt Superoxide (MFI)
1000
800
600
400
200
0
***
Mock
D1
D2
D3
D4
H₂O₂

S2 Fig

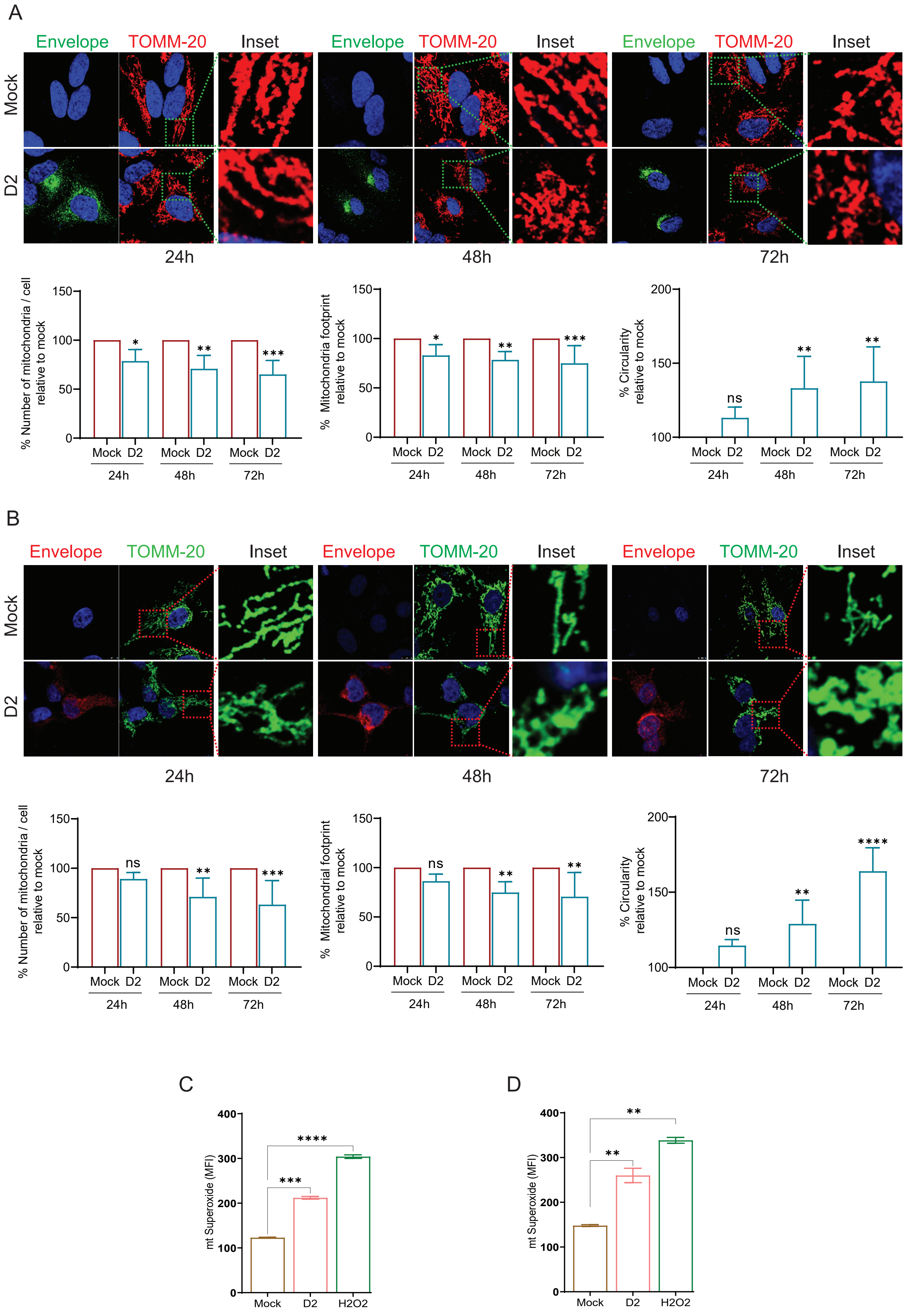
A
Envelope
TOMM-20
Inset
Mock
D2
24h
48h
72h
% Number of mitochondria / cell relative to mock
% Mitochondria footprint relative to mock
% Circularity relative to mock
Mock D2
ns
B
Envelope
TOMM-20
Inset
Mock
D2
24h
48h
72h
% Number of mitochondria / cell relative to mock
% Mitochondrial footprint relative to mock
% Circularity relative to mock
Mock D2
ns
C
mt Superoxide (MFI)
Mock
D2
H2O2
D
mt Superoxide (MFI)
Mock
D2
H2O2

S3 Fig

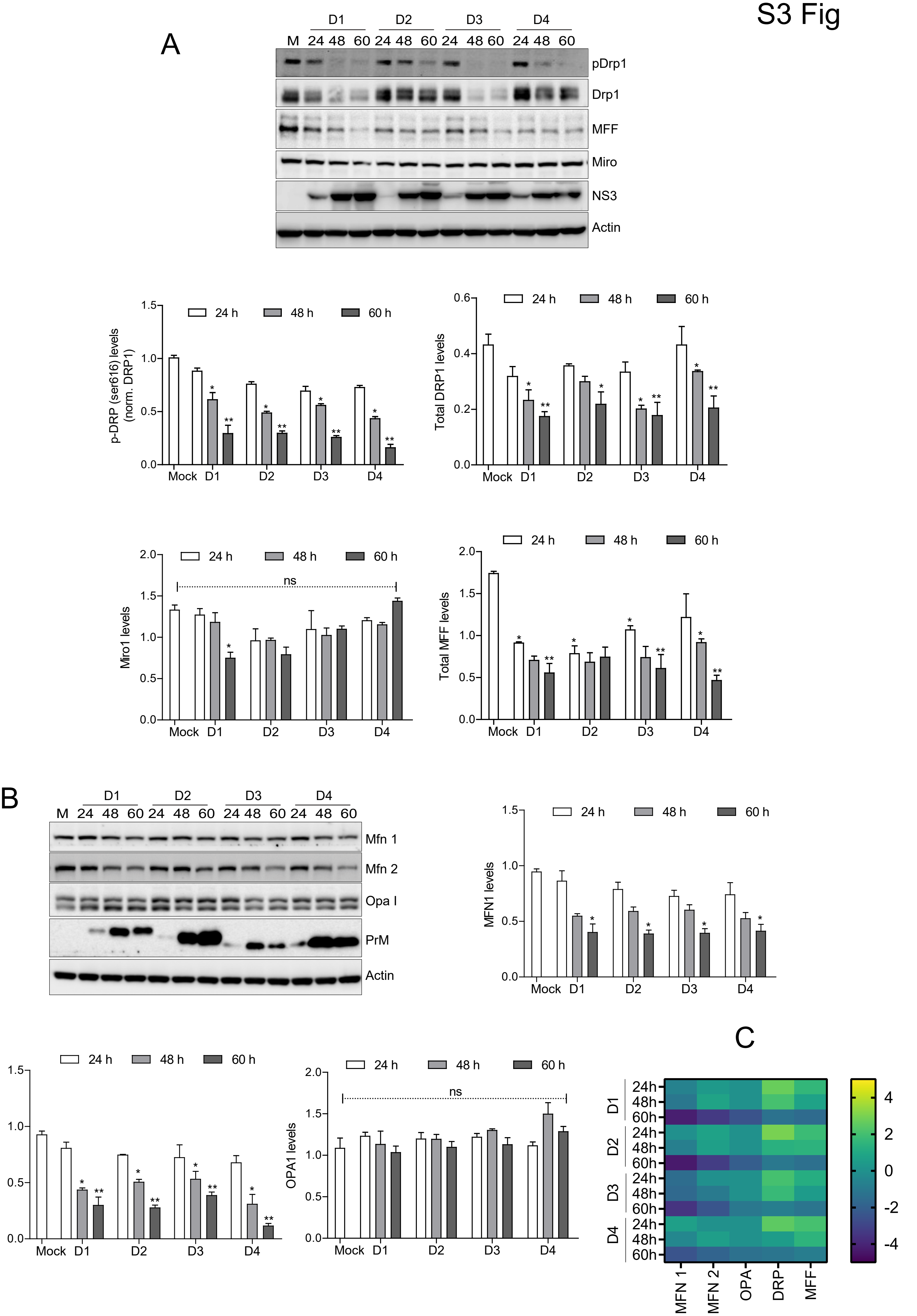

A
D1
D2
D3
D4
M
24 48 60 24 48 60 24 48 60 24 48 60
pDrp1
Drp1
MFF
Miro
NS3
Actin
24 h
48 h
60 h
p-DRP (ser616) levels (norm. DRP1)
Total DRP1 levels
Miro1 levels
Total MFF levels
ns
Mock
B
Mfn 1
Mfn 2
Opa I
PrM
MFN1 levels
MFN2 levels
OPA1 levels
C
24h
48h
60h
MFN 1
MFN 2
OPA
DRP
MFF

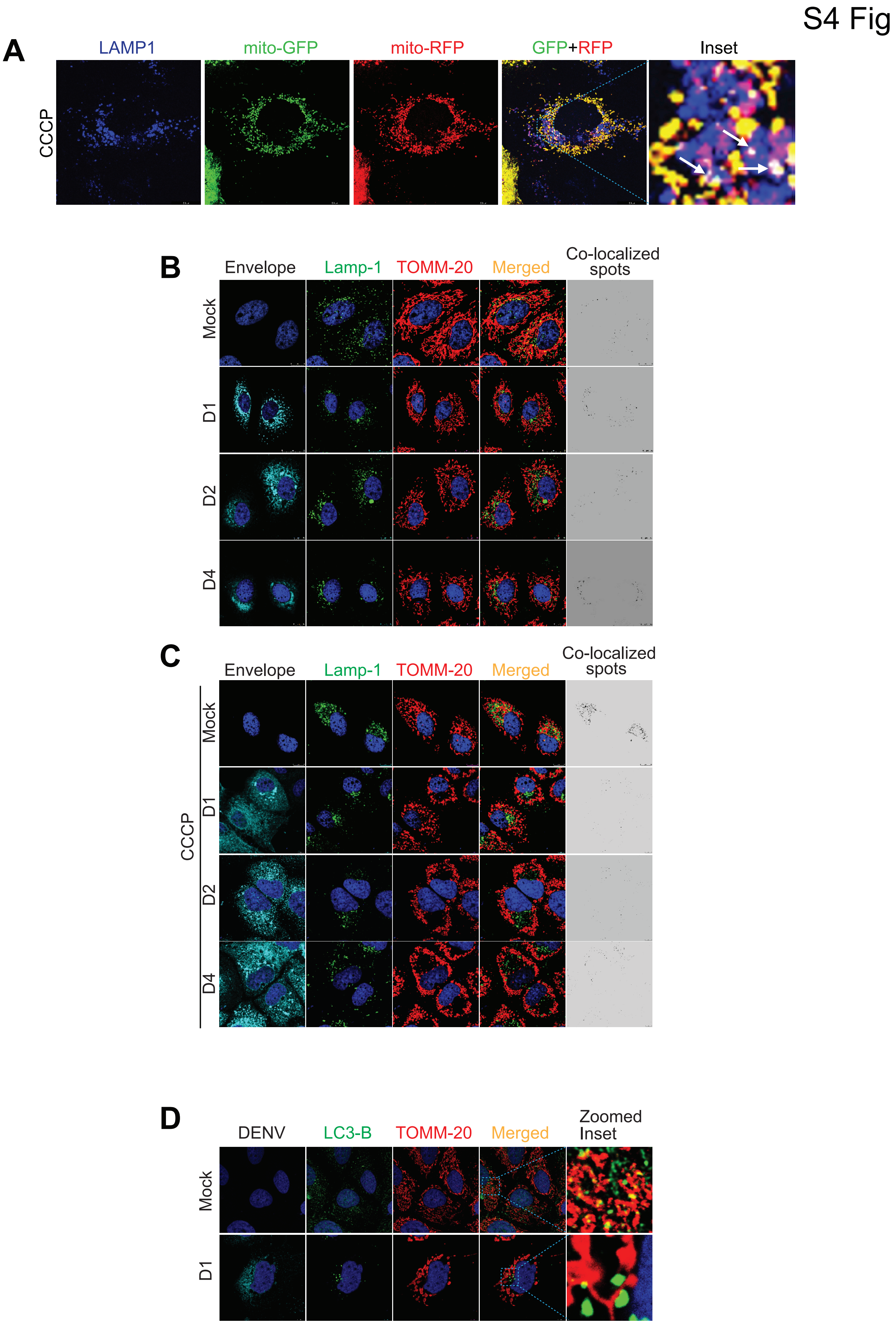
S4 Fig
A
LAMP1
mito-GFP
mito-RFP
GFP+RFP
Inset
CCCP
B
Envelope
Lamp-1
TOMM-20
Merged
Co-localized spots
Mock
D1
D2
D4
C
Envelope
Lamp-1
TOMM-20
Merged
Co-localized spots
CCCP
Mock
D1
D2
D4
D
DENV
LC3-B
TOMM-20
Merged
Zoomed Inset
Mock
D1

S5 Fig

A

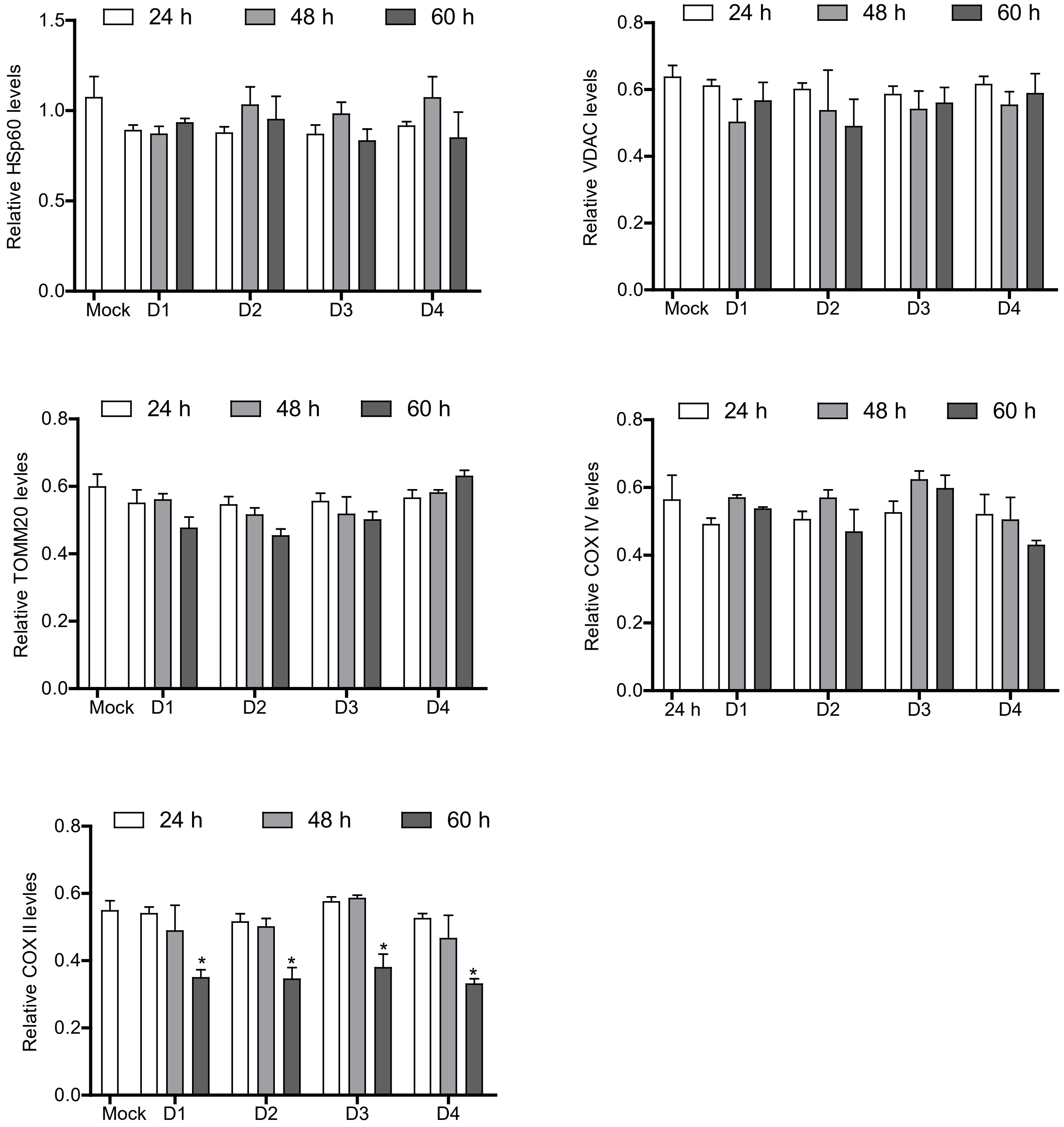
24 h
48 h
60 h
Relative HSp60 levels
1.5
1.0
0.5
0.0
Mock
D1
D2
D3
D4
Relative VDAC levels
0.8
0.6
0.4
0.2
Relative TOMM20 levles
Relative COX IV levles
Relative COX II levles
*

B

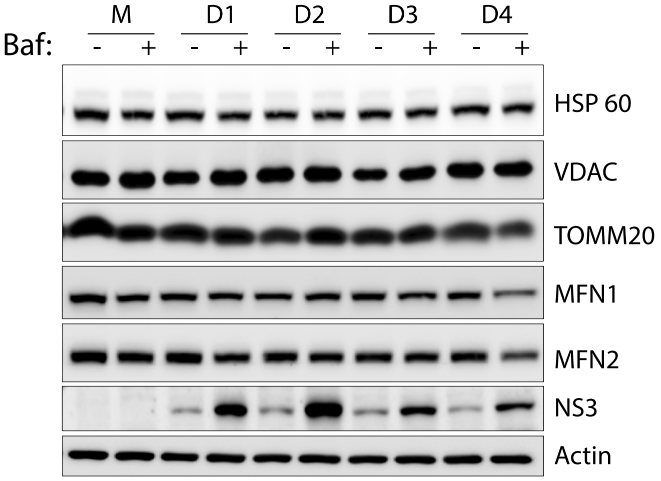
M
D1
D2
D3
D4
Baf:
-
+
HSP 60
VDAC
TOMM20
MFN1
MFN2
NS3
Actin

S6 Fig

A

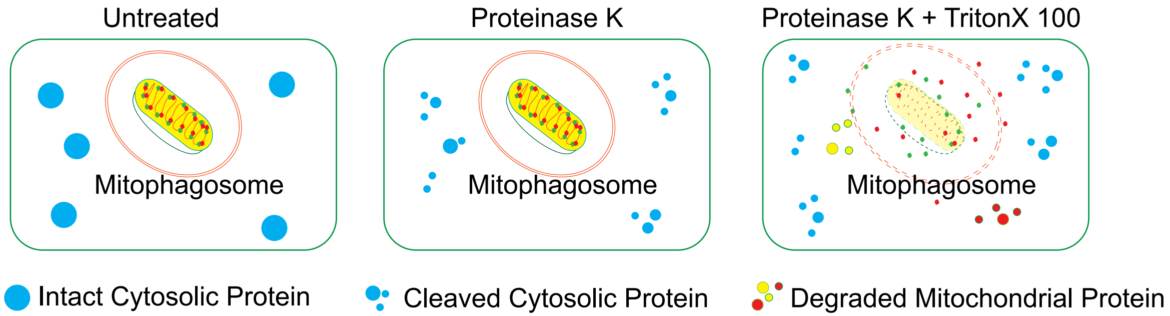

Untreated
Proteinase K
Proteinase K + TritonX 100
Mitophagosome
Mitophagosome
Mitophagosome
Intact Cytosolic Protein
Cleaved Cytosolic Protein
Degraded Mitochondrial Protein

B

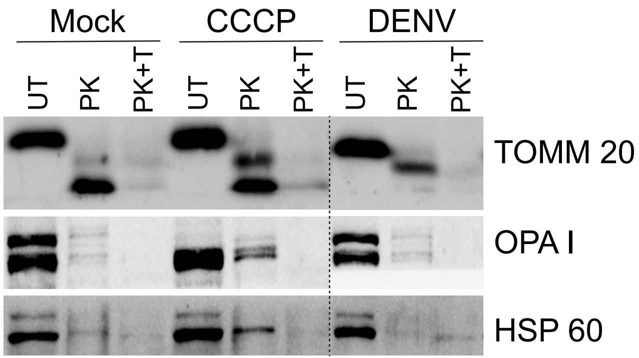

Mock
CCCP
DENV
UT
PK
PK+T
UT
PK
PK+T
UT
PK
PK+T
TOMM 20
OPA I
HSP 60

S7 Fig

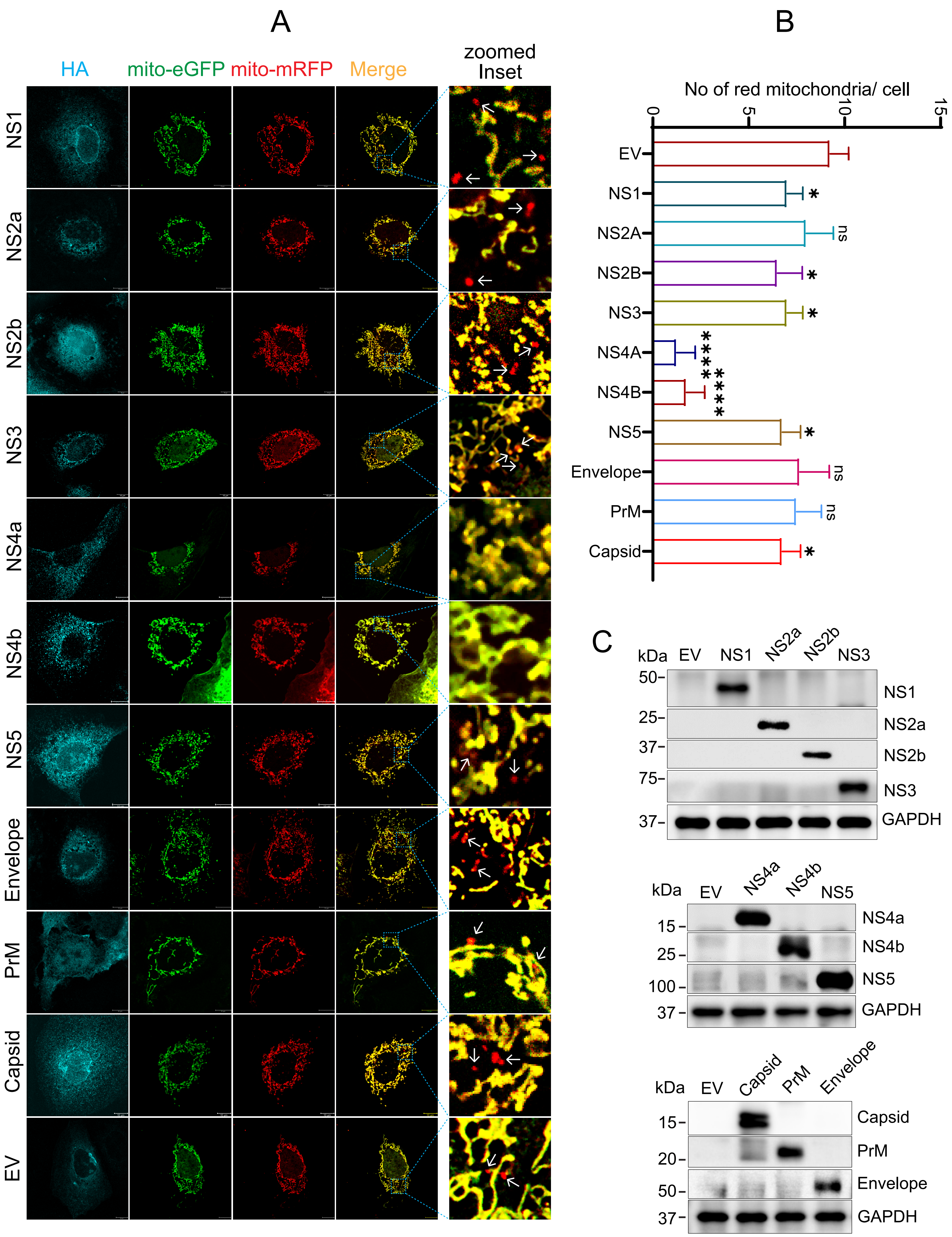
A
HA
mito-eGFP
mito-mRFP
Merge
zoomed Inset
NS1
NS2a
NS2b
NS3
NS4a
NS4b
NS5
Envelope
PrM
Capsid
EV
B
No of red mitochondria/ cell
0
5
10
15
EV
NS1
NS2A
NS2B
NS3
NS4A
NS4B
NS5
Envelope
PrM
Capsid
*
ns
*
*
****
****
*
ns
ns
*
C
kDa
EV
NS1
NS2a
NS2b
NS3
50
25
37
75
37
NS1
NS2a
NS2b
NS3
GAPDH
kDa
EV
NS4a
NS4b
NS5
15
25
100
37
NS4a
NS4b
NS5
GAPDH
kDa
EV
Capsid
PrM
Envelope
15
20
50
37
Capsid
PrM
Envelope
GAPDH

S8 Fig

A

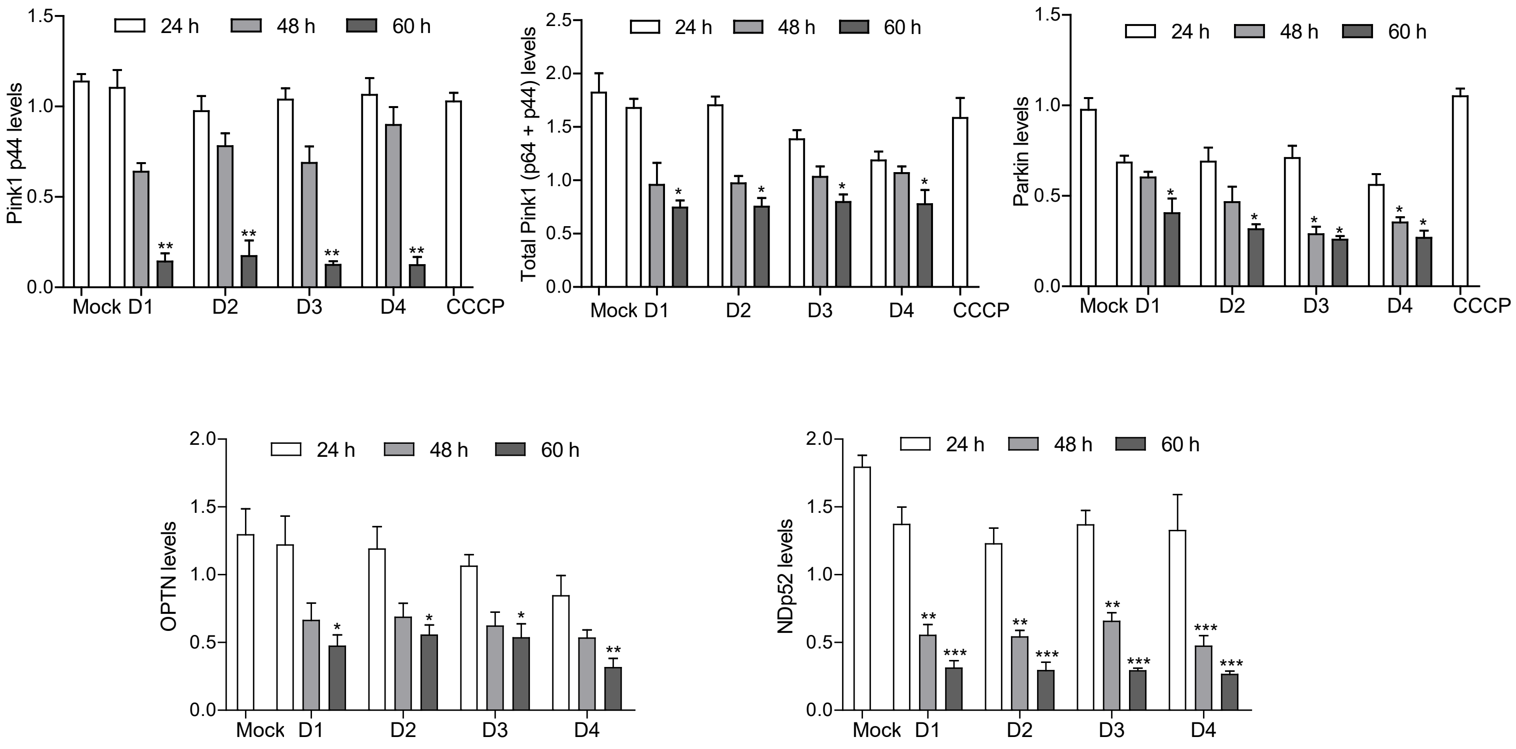
24 h
48 h
60 h
Pink1 p44 levels
Total Pink1 (p64 + p44) levels
Parkin levels
OPTN levels
NDp52 levels
Mock
D1
D2
D3
D4
CCCP

B

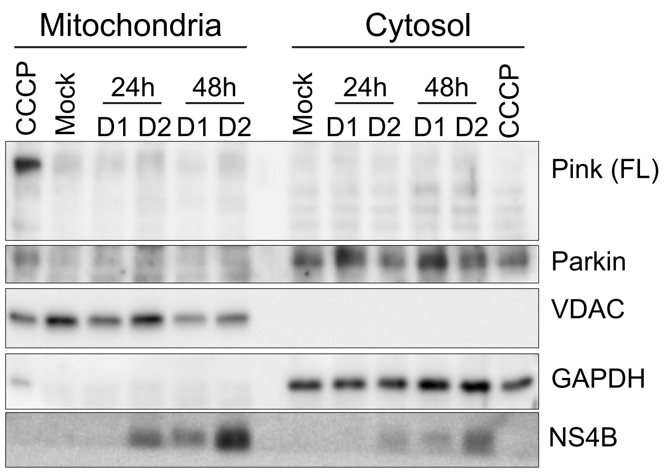
Mitochondria
Cytosol
CCCP
Mock
24h
48h
D1
D2
Pink (FL)
Parkin
VDAC
GAPDH
NS4B

S9 Fig

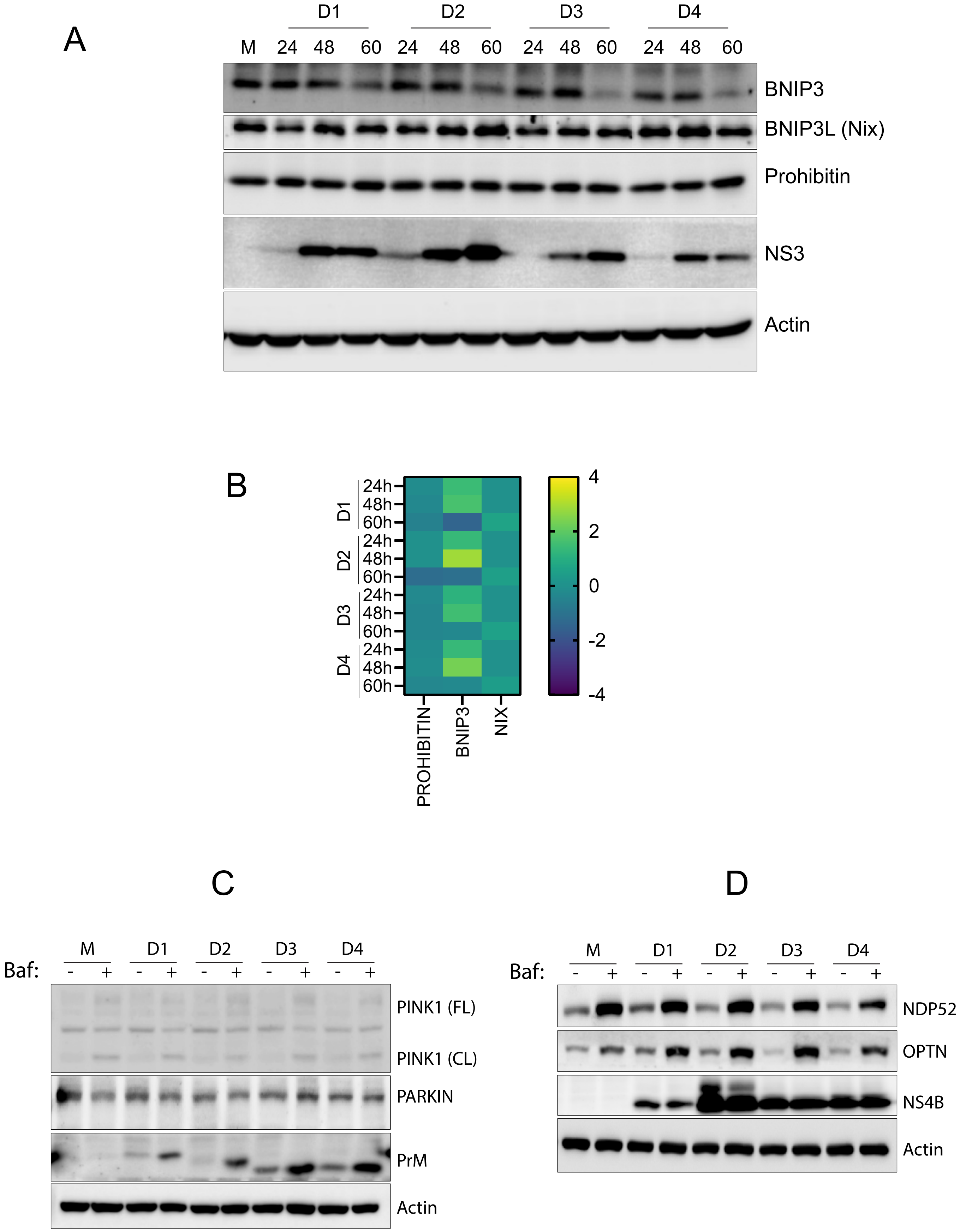
A
D1
D2
D3
D4
M 24 48 60 24 48 60 24 48 60 24 48 60
BNIP3
BNIP3L (Nix)
Prohibitin
NS3
Actin
B
D1
D2
D3
D4
24h
48h
60h
PROHIBITIN
BNIP3
NIX
4
2
0
-2
-4
C
Baf:
M
D1
D2
D3
D4
- +
PINK1 (FL)
PINK1 (CL)
PARKIN
PrM
Actin
D
NDP52
OPTN
NS4B

S10 Fig

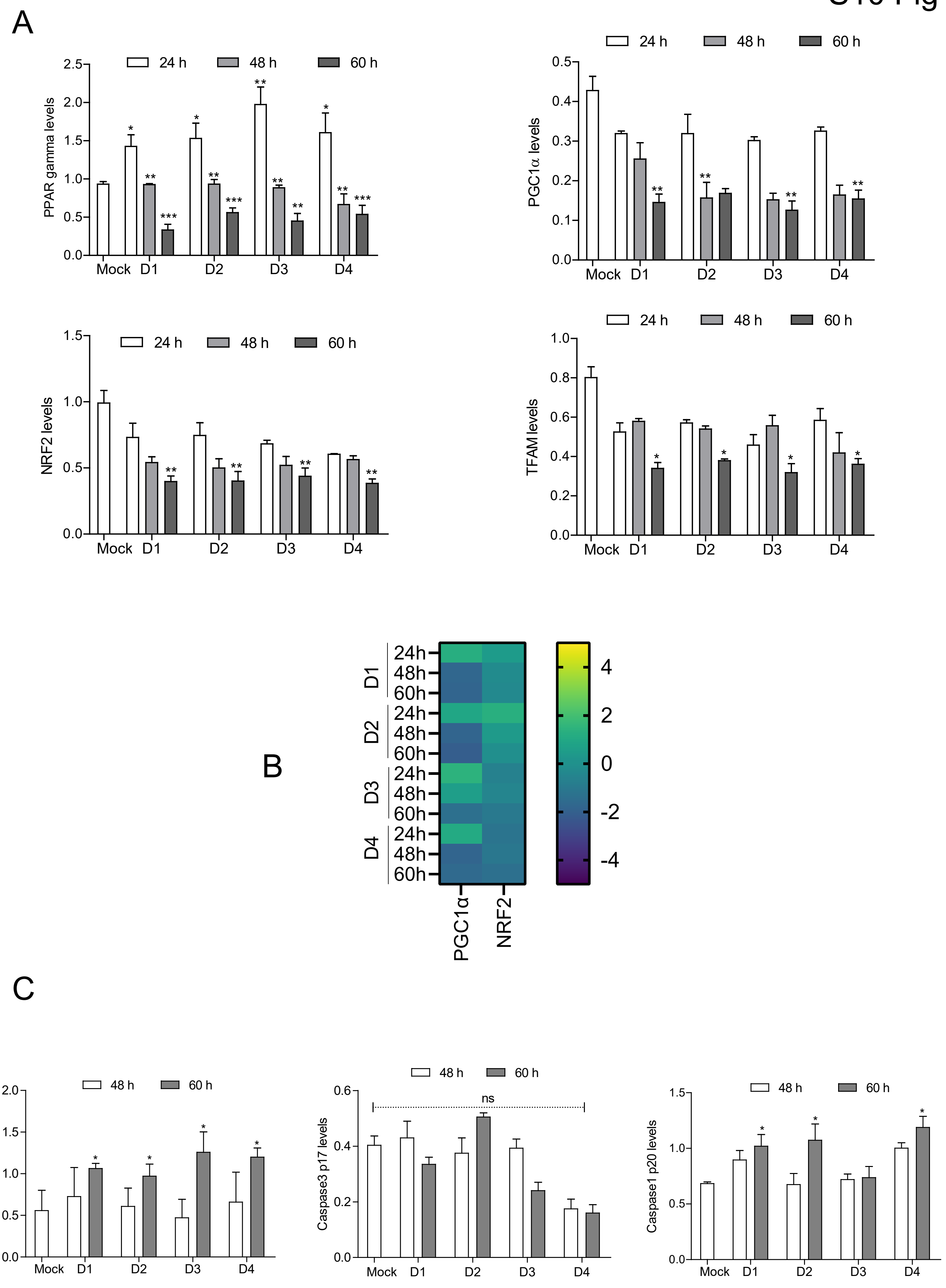

A
24 h
48 h
60 h
PPAR gamma levels
PGC1α levels
NRF2 levels
TFAM levels
Mock
D1
D2
D3
D4
B
D1
D2
D3
D4
24h
48h
60h
PGC1α
NRF2
4
2
0
-2
-4
C
Parp1 p89 levels
Caspase3 p17 levels
Caspase1 p20 levels
ns

S11 Fig

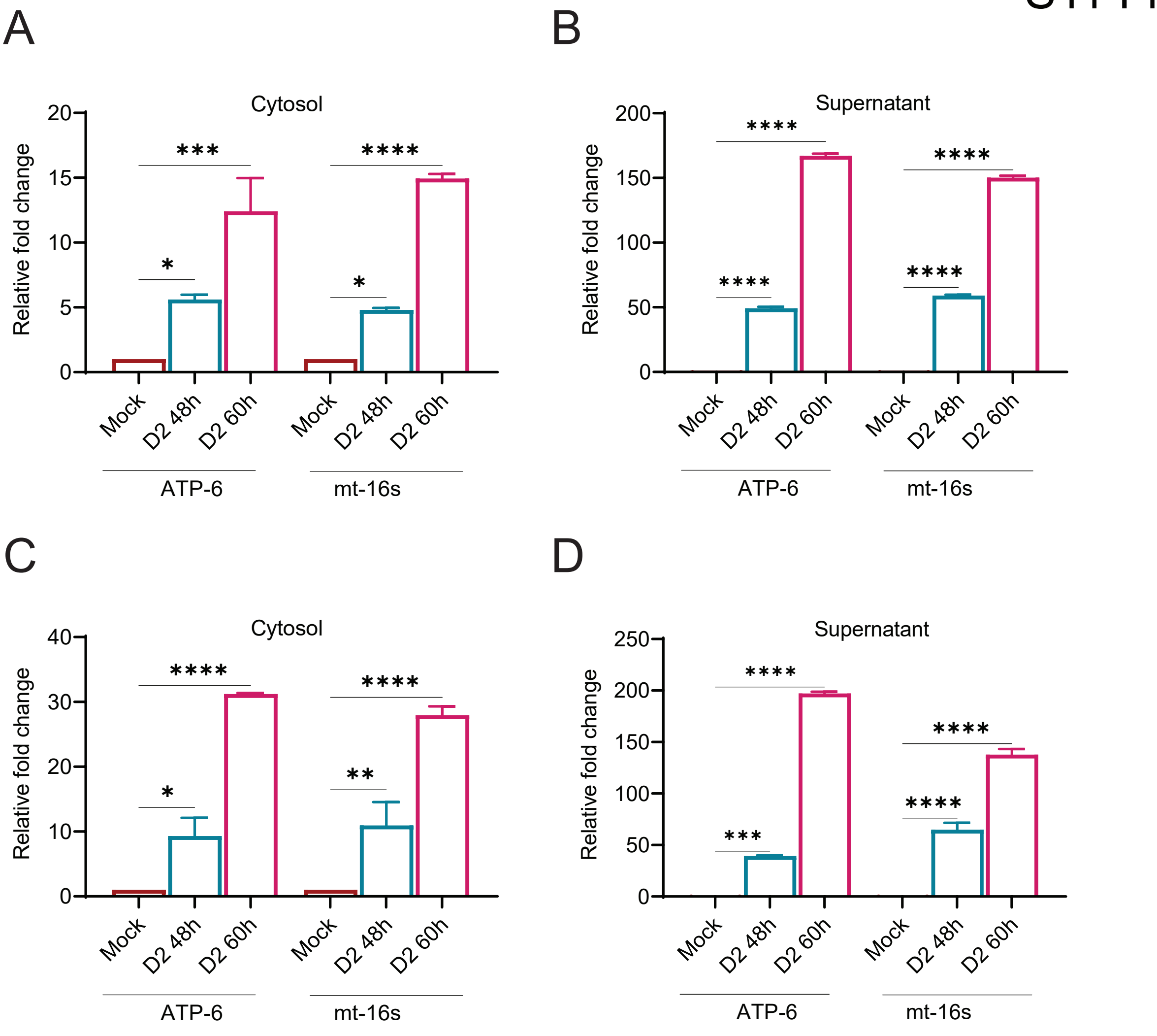
A
Cytosol
Relative fold change
20
15
10
5
0
***
*
****
*
Mock
D2 48h
D2 60h
Mock
D2 48h
D2 60h
ATP-6
mt-16s
B
Supernatant
Relative fold change
200
150
100
50
0
****
****
****
****
Mock
D2 48h
D2 60h
Mock
D2 48h
D2 60h
ATP-6
mt-16s
C
Cytosol
Relative fold change
40
30
20
10
0
****
*
****
**
Mock
D2 48h
D2 60h
Mock
D2 48h
D2 60h
ATP-6
mt-16s
D
Supernatant
Relative fold change
250
200
150
100
50
0
****
***
****
****
Mock
D2 48h
D2 60h
Mock
D2 48h
D2 60h
ATP-6
mt-16s

S12 Fig

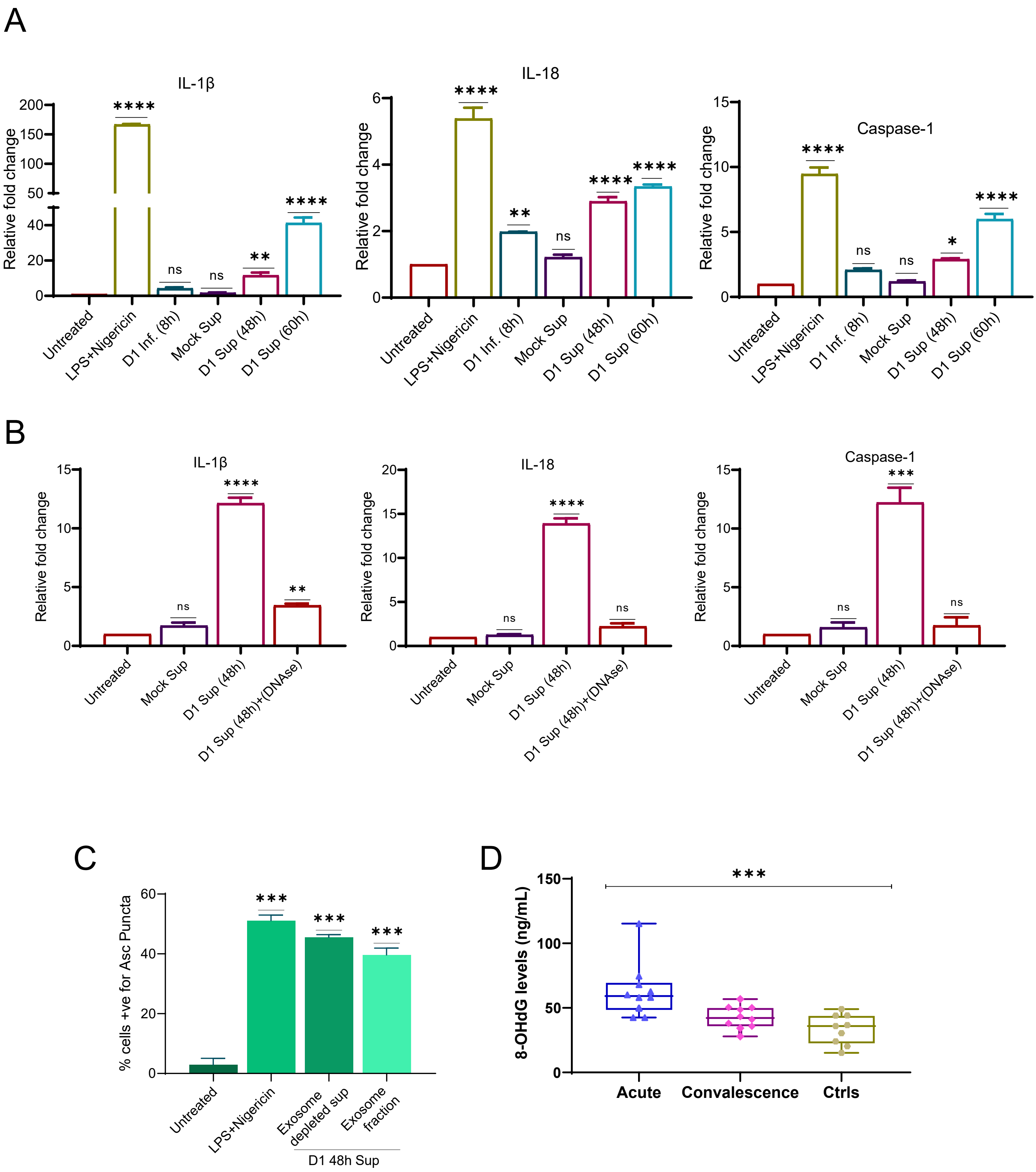

A
IL-1β
Relative fold change
200
150
100
50
40
20
0
****
ns
ns
**
****
Untreated
LPS+Nigericin
D1 Inf. (8h)
Mock Sup
D1 Sup (48h)
D1 Sup (60h)
IL-18
6
4
2
0
****
**
ns
****
****
Caspase-1
15
10
5
0
****
ns
ns
*
****
B
IL-1β
Relative fold change
15
10
5
0
ns
****
**
Untreated
Mock Sup
D1 Sup (48h)
D1 Sup (48h)+(DNAse)
IL-18
20
15
10
5
0
ns
****
ns
Caspase-1
15
10
5
0
ns
***
ns
C
% cells +ve for Asc Puncta
60
40
20
0
***
***
***
Untreated
LPS+Nigericin
Exosome depleted sup
Exosome fraction
D1 48h Sup
D
8-OHdG levels (ng/mL)
150
100
50
0
***
Acute
Convalescence
Ctrls
